# Annulation of a 1,3-dithiole ring to a sterically hindered *o*-quinone core. Novel ditopic redox-active ligands

**DOI:** 10.3762/bjoc.17.26

**Published:** 2021-01-27

**Authors:** Sergey V Norkov, Anton V Cherkasov, Andrey S Shavyrin, Maxim V Arsenyev, Viacheslav A Kuropatov, Vladimir K Cherkasov

**Affiliations:** 1 G. A. Razuvaev Institute of Organometallic Chemistry of Russian Academy of Sciences, Tropinina str. 49, Box-445, 603950 Nizhny Novgorod, Russia; 2 N. I. Lobachevsky Nizhny Novgorod State University, 23 Gagarin Ave, 603950, Nizhny Novgorod, Russia

**Keywords:** dioxolene ligand, 1,3-dithiole, ditopic ligand, o-quinone, thiete

## Abstract

The fused 1,3-dithiole spacer seems to be very suitable for the functionalization of sterically hindered *o*-quinones with additional groups capable of coordination of metal ions and/or possessing a redox activity. An effective method for the synthesis of sterically hindered *o*-quinones containing 1,3-diketonate, dinitrile and *p*-quinone-methide functional groups at the periphery of the ligand has been developed. The novel compounds have rigid and conjugated structures and exhibit properties typical of *o*-quinones. A study of their monoreduced semiquinone derivatives reveal that the spin density is delocalized across the whole molecule, including peripheral fragments. The first stable *o*-quinone derivative bearing an annulated thiete heterocycle has been isolated and characterized.

## Introduction

There are a number of methods which may be used for the synthesis of new *o*-quinones in order to vary their redox properties and coordination abilities. The key features of *o*-quinones are redox activity as well as chelating coordination ability. The redox state of *o*-quinone (semiquinone or catechol) could be changed both as free species or when it is coordinated to a metal ion as a ligand. Thus, the redox isomerism phenomenon was reported for *o*-quinone complexes with both transition and non-transition metals [1–3].

The annulation of sterically hindered *o*-quinones with a 2-substituted 1,3-dithiole cycle seems to be a promising tool for the preparation of redox-active dioxolene species bearing additional functionalities. The previously described *o*-quinones with an annulated 1,3-dithiole fragment display a remarkable stability of different redox states both in non-coordinated form and as being a ligand. It is important that these bicyclic dioxolene species exhibit the typical properties of sterically hindered *o*-quinones, such as redox transformations between *o*-qiunone, semiquinone and catecholate forms as well as coordination ability towards metal ions. The versatile chemistry of precursors used for annulation of the 1,3-dithiole moiety to an *o*-quinone ring allows a wide variety of substituents at the 2-position of the 1,3-dithiole cycle, including additional redox-active, coordination-capable or free-radical functions. *o*-Dioxolene redox-active species bearing additional functions are regarded as promising ligands for the synthesis of metallocomplexes exhibiting unusual magnetic, photovoltaic or luminescent properties. Thus, previously reported binuclear rare-earth metal complexes with redox-active triads such as *o*-quinone-tetrathiafulvalene-*o*-quinone and *o*-quinone-(*p*-phenylene extended tetrathiafulvalene)-*o*-quinone were found to display switching SMM [4–7] or unique luminescent properties [8].

There are many reports describing the preparation of *p*-quinone derivatives annulated with a 1,3-dithiole moiety [9–13], whereas methods resulting in the synthesis of *o*-quinone analogs are scarce.

## Results and Discussion

**Scheme 1:**
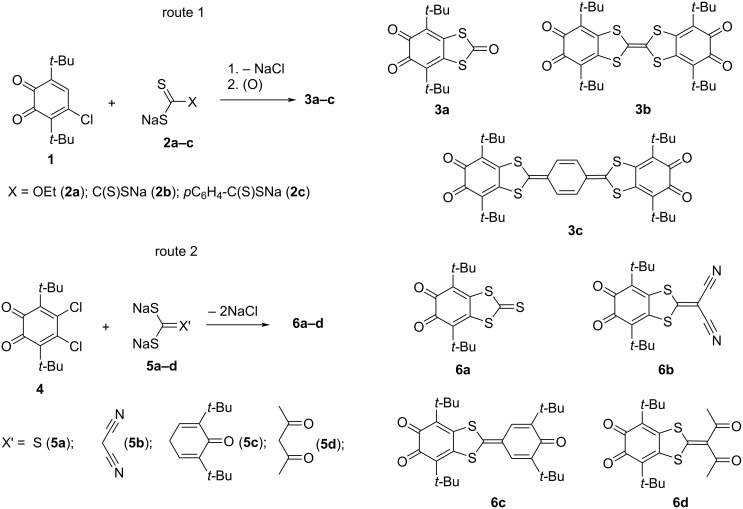
Synthetic pathways for the preparation of *o*-quinone derivatives with annulated 1,3-dithiole ring.

Previously we reported an efficient synthesis pathway for the annulation of the 1,3-dithiole cycle to the 4,5-positions of an *o*-quinone ring using the reaction of 3,6-di-*tert*-butyl-4-chloro-*o*-quinone (**1**) with alkali metal salts of dithiocarboxylic acids. In fact, this procedure is suitable for the synthesis of such annulated *o*-quinone derivatives including di-*o*-quinones bridged by tetrathiafulvalene or *p*-phenylene-extended tetrathiafulvalene spacers **3a–c** (Scheme 1, route 1) [14–17]. Notably, *o*-quinone with an annulated 1,3-dithiole-2-one moiety **3a** was also used as a precursor for the preparation of an *o*-quinone with a fused 1,2-dithiete cycle [18].

Here, we describe another approach to attach the 1,3-dithiole ring to the backbone positions of *o*-quinone. It is the reaction of 4,5-dichloro-3,6-di-*tert*-butyl-*o*-benzoquinone (**4**) [19] with alkali metal *gem*-dithiolates. This synthetic procedure allows us to significantly extend the range of substituents which could be potentially introduced to the final product.

*Gem*-dithiolates are comparatively less studied than related dithiocarboxylates [20–23]. There are some reports in the literature regarding their application as ligands for the preparation of complexes exhibiting non-linear optical activity as well as possessing extraordinary magnetic properties [24]. The simplest structures that were classified both as *gem*-dithiolate and dithiocarboxilate are trithiocarbonate salts.

### Synthesis and characterization of new o-quinone derivatives

**Figure 1:**
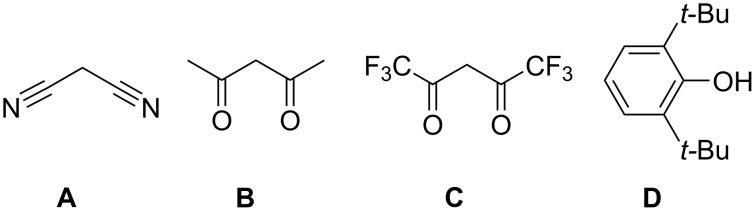
Active methylene compounds used for the preparation of *gem*-dithiolates.

In this work we used sodium *gem*-dithiolates in the reactions with *o*-quinone **4**. Alkali metal *gem*-dithiolates are relatively unstable species, so that they are commonly freshly prepared in situ from the corresponding active methylene compounds (Figure 1) [25]. In our syntheses we also used *gem*-dithiolates immediately, without isolation, except for the case when sodium trithiocarbonate (**5a**) was used.

The reaction of **4** with **5a** proceeds in DMF solution to give the corresponding 1,3-dithiole-2-thione species **6a** in a high yield (Scheme 1, route 2). The red color of the reaction mixture belonging to the parent *o*-quinone rapidly turns into a brown color of *o*-quinone **6a**.

*o*-Quinone **6a** was isolated as red-brown crystals by cooling the acetone/ether mixture. It is a structural analogue of *o*-quinone **3a**, which was previously reported by us [14]. The product **6a** is air-stable at ambient conditions both in solid state and in solution. According to X-ray data, the 1,3-dithiole ring in the molecule is almost planar, its geometry is typical of 1,3-dithiole-2-thione species [26–28] (Figure S1 in Supporting Information File 1). At the same time, the plane of the six-membered *o*-quinone ring is slightly distorted in order to weaken the steric strain effects of the *tert*-butyl groups and neighboring carbonyl oxygens/dithiole sulfur atoms. Such distortion is typical of sterically hindered *o*-quinones and was discussed previously [29].

The reaction of **4** with malononitrile-derived *gem*-dithiolate (**5b**, Scheme 1, route 2) also proceeded under mild conditions in DMF and bicyclic *o*-quinone **6b** was obtained in a good yield. The brown crystals suitable for X-ray analysis were grown from an acetone/diethyl ether mixture.

X-ray diffraction data revealed that the bond lengths distribution in the six-membered ring of **6b** is typical of sterically hindered *o*-quinones. The annulated fragment including the 1,3-dithiole cycle and the malononitrile unit is almost flat. The six-membered *o*-quinone ring is distorted due to the same reasons as discussed above for **6a** (Figure S2 in Supporting Information File 1). The IR spectrum exhibits intense absorption bands related to stretching vibrations of carbonyl groups in *o*-quinones as well as CN functions. ^1^H and ^13^C NMR spectra for *o*-quinone **6b** are also consistent with the structural data.

The reaction of **4** with *gem*-dithiolate **5c** resulted in *o*-quinone **6c** in a good yield (Scheme 1, route 2). TLC control indicated that **6c** was the only colored product in this synthesis.

In *o*-quinone **6c** the six-membered ring at the attached fragment exists in *p*-quinonoid form. According to the X-ray data, this moiety is almost coplanar with the *o*-quinonic unit (Figure S3 in Supporting Information File 1). The double bond connecting the 1,3-dithiole and *p*-quinonoid rings provides a rigid and conjugated structure for the molecule of **6c**. Moreover, due to potential redox activity at the *p*-quinone-methide moiety, compound **6c** should exhibit a more complicated redox behavior compared to parent *o*-quinone.

Despite 1,3-diketones presumably exist as enol tautomers [30], they are also often used as active methylene compounds. The introduction of a 1,3-diketone fragment at the backbone of the *o*-quinone ring seems promising since β-diketones as well as *o*-quinones are known as chelating ligands. The combination of two different chelating sites in the same molecule should give a bifunctional bridging ligand. Such species are interesting from the viewpoint of assembling of ordered structures in a crystal phase.

**Scheme 2:**
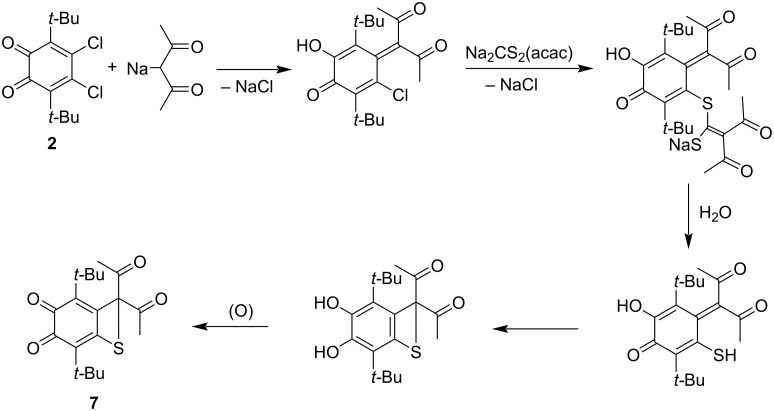
The tentative pathway for the formation of *o*-quinone **7** with annulated thiete ring.

In order to attach the 1,3-diketonate moiety to the periphery of the *o*-quinone molecule, we treated the *o*-quinone **4** with sodium *gem*-dithiolate obtained from acetylacetone (**5d**, Scheme 1, route 2). Unlike interactions with sodium trithiocarbonate or malononitrile *gem*-dithiolate, the *o*-quinone **6d** was not the only product of the reaction of **4** with acetylacetone *gem*-dithiolate **5d**. The final mixture also contained *o*-quinone **7** with attached four-membered thiete ring (Scheme 1 and Scheme 2). The formation of the latter was surprising for us.

The X-ray study of **6d** revealed that the six-membered *o*-quinone exhibit distortion from a mean plane that is typical of sterically hindered *o*-quinones with annulated 1,3-dithiole ring (Figure S4 in Supporting Information File 1). Both carbonyl oxygens of the acetylacetonate unit are directed towards the 1,3-dithiole ring. Such a conformation provides close intramolecular contacts with the sulfur atoms. The S(1)–O(2) distance (Figure S4 in Supporting Information File 1) is 2.560(2) Å, it is less than the sum of the van der Waals radii. A very similar geometry was previously observed in the case of the acetylacetonate unit attached to a 1,3-dithiole ring [31].

*o*-Quinone **7**, which is the minor product in the reaction of **4** with acetylacetone *gem*-dithiolate, is particularly interesting since information on the geometrical configuration of the benzothiete molecule is scarce and, to the best of our knowledge, this is the first example of a structurally characterized benzothiete species. There are some reports concerning benzodithietes, but all of them describe the synthesis by flash pyrolysis technique followed by characterization with NMR spectroscopy [32–33]. Benzothiete is regarded as an isomeric form of *o*-thioquinone methide [34]. A study of the chemical properties of this species is the subject of further investigations.

The bicyclic fragment in **7** is essentially flat (Figure S5 in Supporting Information File 1). The steric interactions between the *tert*-butyl groups and atoms of the fused heterocycle are weaker for the four-membered thiete ring than in the case of the five-membered 1,3-dithiole ring. Less strain effect from the atoms of the annulated ring results in a freer arrangement for *tert*-butyl groups and almost planar geometry for the *o*-quinone cycle, respectively. Such a high planarity of an *o*-quinone unit was also observed in the case of an *o*-quinone with annulated dithiete ring [18].

Due to the ring-strain of the four-membered cycle the bond distance S(1)–C(11) in thiete (1.866(3) Å) is elongated compared to a standard C–S bond lengths in five-membered cycles (≈1.75 Å). The measured value of the C–S–C angle in this ring is 77.4(2)°. Very similar observations were reported previously for monocyclic thiete compounds [35–36]. Benzothietes usually behave as highly reactive and labile species, however, solid samples of *o*-quinone **7** could be stored for months on air at ambient conditions without significant changes. Most probably, such stability of the thiete cycle is a consequence of the protective effect of the bulky *tert*-butyl groups at the quinone ring. *o*-Quinone **7** has a rigid skeleton, the dihedral angle formed by O(1)–C(1)–C(2)–O(2) (*o*-quinone) and O(3)–C(11)–O(4) (acetylacetone) chelating sites mean planes is about 67.4(2)°.

**Figure 2:**
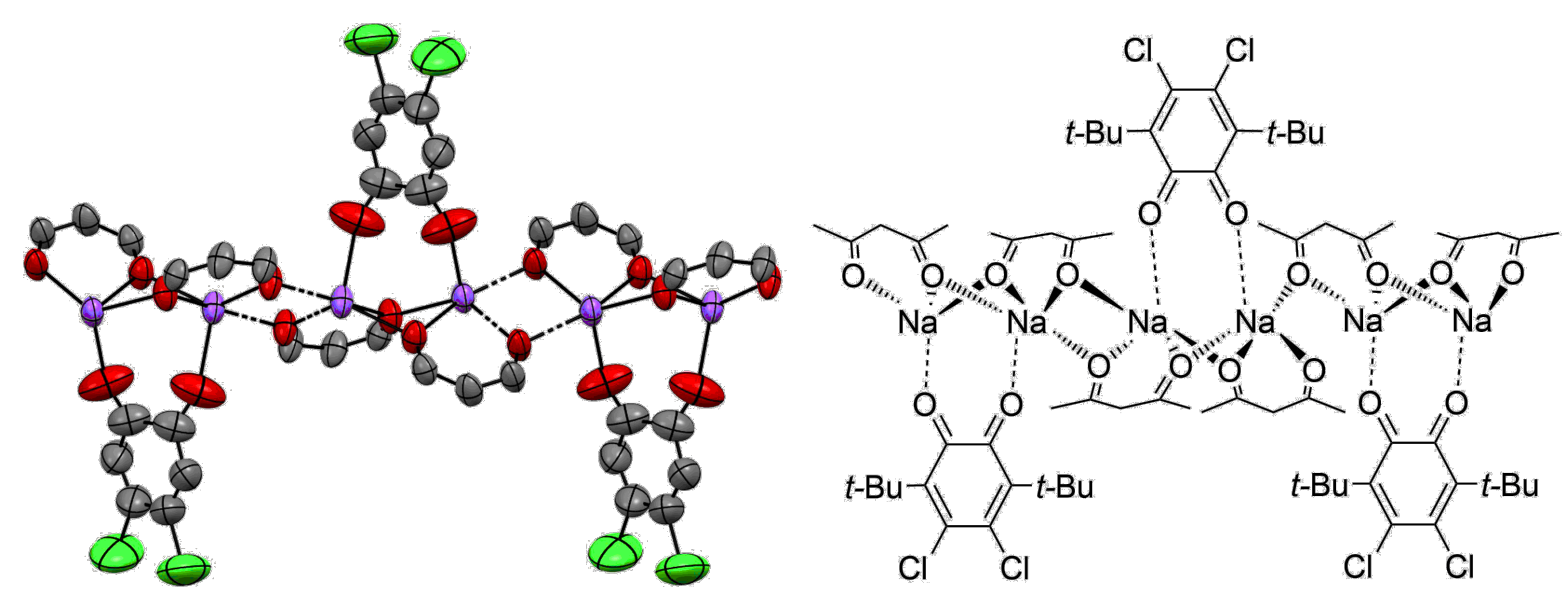
Fragment of coordination polymer chain of adduct **8** in the crystal phase. Hydrogen atoms and CF_3_ groups are omitted for clarity.

In attempt to understand the reasons for the formation of the thiete ring in the reaction with the acetylacetone derivative, we tried to use hexafluoroacetylacetone (hfac) for the preparation of the corresponding sodium *gem*-dithiolate. The mixture of NaH(hfac) with CS_2_ was combined with a solution of **4**. Heating the mixture at 60 °C for 4 h does not result in any change of the color from that characteristic of *o*-quinone **4**. After cooling of the solution red crystals of adduct **8** were isolated (see Figure 2 and Figure S6 in Supporting Information File 1).

The X-ray diffraction study reveals that adduct **8** is a chain coordination polymer, each unit consists of two NaH(hfac) molecules and one molecule of *o*-quinone **4** (Figure 2). The chain is constituted from sodium ions; each sodium ion is surrounded by five oxygens. Four of them belong to hfac molecules, the fifth one is a carbonyl oxygen atom of *o*-quinone **4**. Each *o*-quinone molecule bridges two adjacent sodium ions. The bond lengths distribution in the *o*-quinone moiety in **8** is typical of sterically hindered *o*-quinones (Table S1 in Supporting Information File 1).

According to ^1^H and ^13^C NMR spectroscopy data, adduct **8** in CD_3_OD solution exists as stoichiometric mixture of **4** and monosodium salt of hfac, so that adduct **8** forms in a crystal phase only.

Our studies indicate that the interaction of **4** with stable *gem*-dithiolates, such as sodium trithiocarbonate, malononitrile *gem*-dithiolate or 2,6-di-*tert*-butylphenol *gem*-dithiolate proceeds in a predictable manner to give the corresponding 1,3-dithiole derivatives in a high yield.

On the other hand, the fact that treatment of **4** with NaH(hfac) and CS_2_ does not result in substitution of chlorine atoms at the quinone backbone indicates that under such conditions the *gem*-dithiolate species is not formed. Moreover, the nucleophilicity of the hexafluoroacetylcetonate anion is also insufficient for the substitution of chlorine atoms at the *o*-quinone backbone.

In the case of the reaction of **4** with acetylacetone *gem*-dithiolate **5d** the tentative mechanism of the formation of thiete species **7** is depicted in Scheme 2. The high extent of enolization of 1,3-diketones results in lower nucleophilicity of the active methylene function. This fact enhances the reversibility of the dithiocarboxylation of sodium acetylacetonate. It is known that dithiocarboxylates and *gem*-dithiolates can lose CS_2_ resulting in active methylene compound salts [17]. Due to the reversibility of dithiocarboxylation, the reaction mixture contains some amount of sodium acetylacetonate, which can attack the chlorine atom at the backbone of **2** to give the substitution product (Scheme 2). According to the reported data, the quinone-methide form is more preferable for such species than the *o*-quinone one [37]. After the first step, the remaining chlorine atom in the molecule could not be a subject for attack from another molecule of sodium acetylacetonate since it is shielded by neighboring groups, but it could be substituted with the less bulky sulfur-containing group. The possible pathway for the following cyclization is the Michael addition of the sulfide atom to the central carbon of the acetylacetonate group [38–41].

The X-ray diffraction study as well as IR and NMR spectroscopy data reveal that in all synthesized compounds **6a–d** and **7** the dioxolene moiety exhibits structure features which are typical of sterically hindered *o*-quinones. We conducted some reactions in order to characterize these new species.

**Scheme 3:**
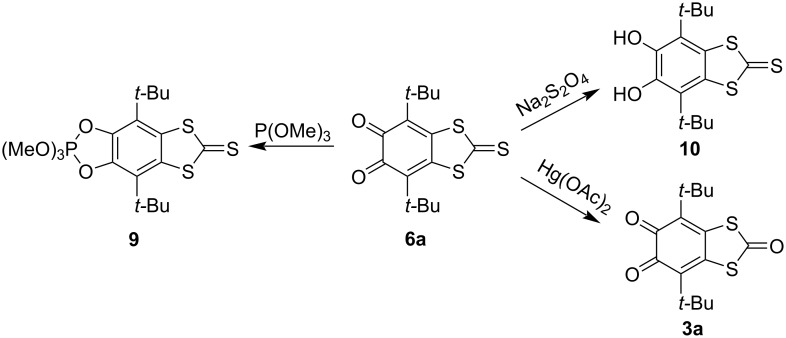
Reactions of *o*-quinone **6a**.

Recently we reported that **3a**, which is the carbonyl analogue of **6a**, being treated with more than a triple excess amount of P(OMe)_3_ forms only the dioxaphospholane species, whereas the periphery carbonyl function remained unchanged [14]. *o*-Quinone **6a** behaves in a similar manner: interaction with an excess amount of P(OMe)_3_ results in the formation of dioxaphospholane derivative **9** (Scheme 3, Figure S7 in Supporting Information File 1). Taking into account the selectivity and almost quantitative yield of this reaction, such a phosphorylation seems to be useful for the protection of the dioxolene site in case of necessity to modify the periphery of the molecule.

The thiocarbonyl group in **6a** also remained unchanged upon reduction with sodium dithionite in a water/methanol mixture: at these conditions *o*-quinone **6a** converts into catechol **10**. It has been shown also that the thiocarbonyl group in **6a** could be easily converted into a carbonyl group by action of mercury(II) acetate in solution (Scheme 3).

### EPR spectroscopy studies

The key purpose of this work is the synthesis of species bearing both an *o*-dioxolene coordination site and an additional peripheral function capable of binding to a metal ion. The study of coordination abilities of peripheral groups will be the subject of further investigations. In this work we have tested only typical properties of the dioxolene function of the novel synthesized species.

**Scheme 4:**
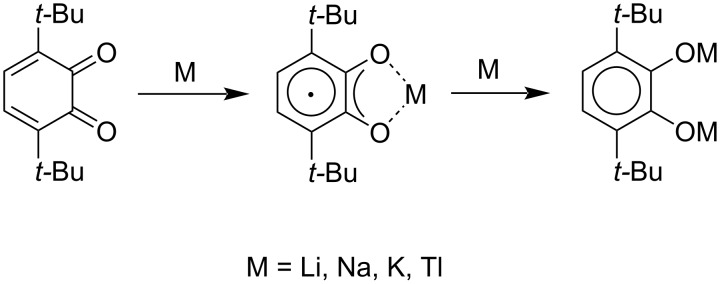
Stepwise reduction of *o*-quinones with metals to semiquinonates and catecholates, respectively.

As mentioned above, all new *o*-quinones **6a–d** and **7** are stable at rt on air both in crystalline form and in solution. Like other sterically hindered *o*-quinones, they can undergo two consecutive one-electron reductions in solution being treated with an alkali metal or thallium amalgam yielding in semiquinonate and then the catecholate species, respectively (Scheme 4). The corresponding semiquinonates could be also generated in a solution in the reaction with Mn_2_(CO)_10_ under irradiation with visible light. The semiquinonate species were studied in solution by EPR spectroscopy (Figures S8–S25 in Supporting Information File 1); the parameters of their isotropic EPR spectra are tabulated in Table S4 in Supporting Information File 1. The measured values of the splitting constants were fitted using the computer simulation of the experimental EPR spectra.

It is noteworthy that semiquinone derivatives of **6b** and **6d** show resolved hyperfine splittings due to the coupling of unpaired electrons with the magnetic nuclei of ^14^N (quintet 1:2:3:2:1) and protons of methyl groups (septet 1:6:15:20:15:6:1), respectively. *o*-Semiquinones are often used as convenient spin labels to determine the spatial arrangement of ligands and to monitor the processes in the coordination sphere of metal ions in real time [42–46]. The spin density distribution and thus the hyperfine coupling constants for the magnetic nuclei at the semiquinone molecule usually are highly dependent on the surrounding at the chelating site of the ligand. In case of ditopic *o*-quinones **6b** and **6d** the parameters of hyperfine splitting on these magnetic nuclei should depend on the coordinated/non-coordinated state of both sites.

Semiquinone derivatives of **7** yield EPR signals of the same multiplicity as they were observed in the case of spectra of monoreduced species of **6d**, but the value of hyperfine splitting constant on protons of the methyl groups of the acetylacetonate fragment (0.17 G for potassium semiquinonate, see Table S4 in Supporting Information File 1) is less than that in **6d** (0.31 G for potassium semiquinonate). This could be explained by the fact that unlike **6d**, the acetylacetonate fragment in **7** lies in an almost orthogonal plane relative to the semiquinone ring that disfavors delocalization of the spin density on the methyl groups.

The EPR spectroscopy study of the semiquinonate species of *o*-quinone **6c** also confirms the delocalization of the spin density onto the periphery of the ligand. Hyperfine splitting constants with the protons of the *p*-phenylene ring were observed in the EPR spectra in solution (Table S4 in Supporting Information File 1). The values of coupling constants with these protons vary in the range of 0.18–0.30 G, they are highly sensitive to the nature of the metallofragment at the chelating dioxolene site. With metals tending to high ionicity in the bonding with the ligand we observed smaller values of coupling constants.

*o*-Quinones **6a**, **6b**, **6d** and **7** have a rigid and almost planar structure, moreover, they bear an additional coordination site at the periphery of the molecule potentially capable of binding of metal ions. Such a topology of the ligands is promising from the viewpoint of its use in the design of coordination polymers. A possibility for spin density delocalization across *o*-quinone and annulated moieties, found by EPR spectroscopy study of their monoreduced derivatives, is a confirmation for the hypothesis that such ditopic ligands could provide an effective channel for electronic exchange interactions between the metal ions localized at different sites of the molecule.

### Electrochemistry studies

Cyclic voltammograms for new *o*-quinones have been recorded in CH_3_CN solution containing 0.10 M (*n*-Bu_4_N)ClO_4_ as supporting electrolyte at a glassy carbon working electrode and a Ag/AgCl/KCl reference electrode. Table 1 summarizes the results of the electrochemical studies.

**Table 1:** Cyclovoltammetry data for o-quinones.

Quinone	*E*^1^_1/2_ Red (V)	*E*^2^_1/2_ Red (V)	*E*^3^_1/2_ Red (V)	*E*^4^_1/2_ Red (V)

**36Q**	−0.50	−1.10	–	–
**3a**	−0.32	−0.95	–	–
**6a**	−0.27	−0.88	–	–
**6b**	−0.17	−0.89	−1.85	–
**6c**	−0.16	−0.37	−0.88	−1.64
**6d**	−0.31	−1.73	1.92	–
**7**	−0.49	−1.24	–	–

Usually, the electrochemistry of sterically hindered *o*-quinones is relatively simple, it includes two one-electron steps with separation of potentials. Cyclic voltammetry studies reveal that the reduction of **3a**, **6a** and **7** takes place in two subsequent one-electron quasi-reversible steps corresponding to the formation of semiquinone and dianion catecholate species, respectively

*o*-Quinones **6b**–**d** bearing an additional redox-active unit in the structure exhibit a more complicated behavior that involve processes on the annulated moiety. The reduction potential values of 2-substituted malononitriles and 2-substituted acetylacetones are usually higher than those of o-quinones, so that in the case of **6b** and **6d** two first distinct waves could be attributed to subsequent reductions of the dioxolene fragment. Further reductions correspond to processes on the peripheral functions.

*o*-Quinone **6c** exhibits four one-electron quasi-reversible reduction waves. This compound contains of two redox-active fragments – 3,6-di-*tert*-butyl-*o*-quinonoid and 2,6-di-*tert*-butyl-*p*-quinone-methide, respectively. According to the literature data they should have very similar values of first reduction potentials [47–48]. We assume that since the molecule of **6c** has an almost planar skeleton and conjugated π-system, the observed redox-processes could not be attributed with confidence to a particular unit of the molecule.

It should be mentioned, for all studied *o*-quinone species with annulated 1,3-dithiole fragment, an increasing oxidizing ability comparing to that of 3,6-di-*tert*-butyl-*o*-benzoquinone (**36Q**) (*E*_red_(1/2) = −0.50 V) was observed. For *o*-quinones **6a–d** the first reduction potential is shifted by 0.18–0.32 V relative to that of **36Q**. Previously reported *o*-quinone **3b** was also found to be a stronger oxidant than **36Q** [16]. A very similar redox behavior and shifts of reduction potential values were observed for *p*-quinone derivatives with attached 1,3-dithiole rings [27,49].

### UV–vis spectroscopy

Sterically hindered *o*-quinones are deep-colored compounds. Any changes in their redox or/and coordination state are accompanied by changes in UV–vis spectra. For compounds **3a, 6a–d** and **7** UV–vis spectra were recorded in CH_3_CN (see Supporting Information File 1 and Table 2). It is noteworthy that *o*-quinones with annulated 1,3-dithiole group seem to be more even highly colored species than **36Q**. Compounds **3a** and **6a** exhibit two-bands in the UV–vis spectra those are typical of sterically hindered *o*-quinones. Taking into account the previously studied spectra of substituted derivatives of **36Q**, an intensive short-wave band could be assigned to the n–π* transition, whereas the wide long-wave band corresponds to the π–π* transition [50].

**Table 2:** UV–vis absortption spectra data of *o*-quinones.

Quinone	Wavelength, nm (ε, mol^−1^·L·cm^−1^)		

**36Q**	–	415 (2500)	584 (100)
**3a**	–	445 (2000)	585 (570)
**6a**	–	395 (11200)	600 (320)
**6b**	305 (9300)/ 320 (9400)	400 (10300)	567 (600)
**6c**	390 (22200)	404 (23000)	512 (12400)
**6d**	318 (8600)	425 (13400)	588 (500)
**7**	306 (6400)	447 (3400)	564 (400)

UV–vis spectra of **6b**–**d** are more complicated and show the bands characteristic of both *o*-quinone and an attached fragment. So that *o*-quinone **6c** displays absorptions typical of 1,3-diketones [51]. *o*-Quinone **6b** exhibits intensive absorption peaks in the 300–400 nm region. Very similar bands were previously observed for *p*-quinone malononitrile derivatives [11].

## Conclusion

In conclusion, a new and effective synthesis pathway to chelating ditopic ligands – 3,6-di-*tert*-butyl-*o*-benzoquinones annulated at the 4,5-positions with a 2-substituted 1,3-dithiole ring – has been developed. A series of new *o*-quinones bearing additional coordination sites at the periphery of the molecule have been prepared. Structural studies reveal a rigid and almost planar structure for these functionalized *o*-quinones, such topology is promising since potentially it can be useful in the design of coordination polymers. A stable *o*-quinone derivative containing an annulated thiete ring was isolated and characterized. The use of these new ligands for the preparation of binuclear complexes or coordination polymers seems to be interesting from the viewpoint of construction of molecular devices, since according to the EPR data for its monoreduced derivatives, there is a channel for the intraligand electron exchange interaction between the coordination sites of the molecule.

## Experimental

All chemicals and solvents were purchased from commercial sources. Solvents were purified in standard ways. Experimental procedures for the synthesis of compounds **3a**, **6a**–**d**, **7**–**10** are described in Supporting Information File 1. The infrared spectra of compounds in the 4000–400 cm^−1^ range were recorded on FSM 1201 Fourier-IR spectrometer in nujol. NMR spectra were recorded in CDCl_3_ solution by using a Bruker Avance III 400 MHz instrument with TMS as an internal standard. EPR spectra were obtained using a Bruker EMX CW X‐band EPR spectrometer. Cyclic voltammetric measurements for new *o*-quinones were performed in MeCN in argon atmosphere on the Elins P-45X instrument using a glassy carbon working electrode (2 mm diameter), platinum wire counter electrode and Ag/AgCl/KCl reference electrode.

### X-ray crystallography

The X-ray data for **6a–d** and **7–9** were collected on Rigaku OD Xcalibur (**6b**–**d, 7, 9**) and Bruker D8 Quest (**6a**, **8**) diffractometers (Mo Kα radiation, ω-scans technique, λ = 0.71073 Å) using APEX3 [52] and CrysAlis^Pro^ [53] software packages. The structures were solved by direct methods and were refined by full-matrix least squares on *F^2^* for all data using SHELX [54]. SADABS [55] and CrysAlis^Pro^ were used to perform the absorption corrections. All non-hydrogen atoms in **6a**–**d** and **7**–**9** were found from Fourier syntheses of electron density and were refined anisotropically. All hydrogen atoms were placed in calculated positions and were refined in the “riding” model with *U(H)*_iso_ = 1.2*U*_eq_ of their parent atoms (*U(H)*_iso_ = 1.5*U*_eq_ for methyl groups). **6c** and **7** were refined as inversion (**6c**) and non-merohedral (**7**) twins with ratios 0.49 and 0.34, respectively.

The crystallographic data and structure refinement details for **6a**–**d** and **7**–**9** are given in Table S2 in Supporting Information File 1. CCDC–1937741 (**6a**), 1937742 (**6b**), 1952206 (**6c**), 1937743 (**6d**), and 1937744 (**7**), 2013491 (**8**) and 1986138 (**9**) contains the supplementary crystallographic data for this paper. These data are provided free of charge by The Cambridge Crystallographic Data Centre: http://www.ccdc.cam.ac.uk/structures.

### Samples for EPR spectroscopy

Alkali metal, thallium, hydrogen semiquinonates as well as manganese carbonyl semiquinonates from *o*-quinones **3**, and **7**–**10** were generated directly before recording of EPR spectra according to the procedure described previously [56–57].

## Supporting Information

File 1 Experimental and analytical data.
