# Supplementary material for: Annulation of a 1,3-dithiole ring to a sterically hindered o-quinone core. Novel ditopic redox-active ligands
Source: Beilstein J Org Chem. 2021 Jan 27;17:273–82. doi: 10.3762/bjoc.17.26 (PMC7849243; doi:10.3762/bjoc.17.26)
Supplement: File 1 — Experimental and analytical data. [file Beilstein_J_Org_Chem-17-273-s001.pdf]

BEILSTEIN JOURNAL OF ORGANIC CHEMISTRY

Supporting Information

for

Annulation of a 1,3-dithiole ring to a sterically hindered *o*-quinone core. Novel ditopic redox-active ligands

Sergey V. Norkov, Anton V. Cherkasov, Andrey S. Shavyrin, Maxim V. Arsenyev, Viacheslav A. Kuropatov and Vladimir K. Cherkasov

Beilstein J. Org. Chem. **2021,** *17,* 273–282. doi:10.3762/bjoc.17.26

Experimental and analytical data

License and Terms: This is a supporting information file under the terms of the Creative Commons Attribution License (https://creativecommons.org/licenses/by/4.0). Please note that the reuse, redistribution and reproduction in particular requires that the author(s) and source are credited and that individual graphics may be subject to special legal provisions.
The license is subject to the *Beilstein Journal of Organic Chemistry* terms and conditions: (https://www.beilstein-journals.org/bjoc/terms)

Contents

1. Procedures for synthesis of compounds	S2
2. X-ray diffractometry data	S5
3. EPR spectroscopy data and EPR spectra	S13
4. Electrochemistry data	S19
5. UV–vis spectra	S22
6. NMR spectra	S25

Procedures for synthesis of compounds

4,7-Di-*tert*-butyl-2-thioxobenzo[*d*][1,3]dithiole-5,6-dione (**6a**) Solution of sodium tritiocarbonate (1.39 g, 9 mmol) in 15 mL DMF was added to the solution 3,6-di-*tert*-butyl-4,5-dichloro-*o*-quinone **2** (2.60 g, 9 mmol) in 15 mL of DMF dropwise at 0 °C. Then the mixture was allowed to warm to room temperature and the color of the solution immediately turned from red to red-brown. After that the mixture was stirred for another 15 minutes, and then the solution is poured into cool water. The product was extracted from an aqueous solution with diethyl ether (5 × 45 mL). The ether solution is washed with water (5 × 50 mL) and then solvent was removed on a rotary evaporator. The product is recrystallized from acetone-diethyl ether (1: 3) mixture at −18 °C. Red-brown crystals. (2.10 g, 73%); m.p. 140 °C; Anal. Calcd for $C_{15}H_{18}O_2S_3$: C, 55.18; H, 5.56; S, 29.46. Found: C, 55.94; H, 5.40; S, 30.21; ^{1}H NMR (CDCl$_3$, 400 MHz): δ 1.40(s, 18H); ^{13}C{1H} NMR (CDCl$_3$, 100 MHz): δ 30.1, 37.9, 141.6, 153.1, 184.6, 210.3; IR (cm^{-1}): 1665, 1634, 1395, 1365, 1290, 1267, 1218, 1164, 1094, 1082, 1026, 989, 914, 835, 816, 764, 692, 627, 592, 528

4,7-Di-*tert*-butyl-2-oxobenzo[*d*][1,3]dithiole-5,6-dione. (3a) Hg(OAc)$_2$ was added to the solution 4,7-di-*tert*-butyl-2-thioxobenzo[*d*][1,3]dithiole-5,6-dione (326 mg, 1 mmol) in 30 mL of mixture methanol-acetic acid (1:1) and stirred for 4 h at rt. Then the mixture was filtered and solvent was removed on a rotary evaporator. The product is recrystallized from diethyl ether at −18 °C. (198 mg, 64%); m.p. 84-85 °C; Anal. Calcd for $C_{15}H_{18}O_3S_2$: C, 58.04; H, 5.84; S, 20.66. Found: C, 57.96; H, 5.77; S, 20.93; ^{1}H NMR (CDCl$_3$, 400 MHz): δ 1.37 (s, 18H); ^{13}C{1H} NMR (CDCl$_3$, 100 MHz): δ 30.1, 37.7, 144.7, 146.1, 186.3, 210.3; IR (cm^{-1}): 1832, 1755, 1734, 1652, 1600, 1507, 1393, 1360, 1285, 1222, 1083, 1028, 928, 832, 685, 648, 575

2-(4,7-Di-*tert*-butyl-5,6-dioxo-5,6-dihydrobenzo[*d*][1,3]dithiole-2-ylidene)malononitrile (**6b**) Malononitrile (132 mg, 2 mmol) in 10 mL of methanol was added dropwise at vigorous stirring to sodium methoxide (108 mg, 2 mmol) solution in methanol (5 mL) and then stirred for 30 minutes. Then carbon disulfide (152 mg, 2 mmol) was slowly added dropwise and stirred for another 30 minutes. After that another portion of sodium methoxide (108 mg, 2 mmol) in 5 mL of methanol was slowly added and then stirred also for 30 minutes. Finally, the color of the solution turned to a light yellow. A solution of 3,6-di-*tert*-butyl-4,5-dichlorocyclohex-3,5-diene-1,2-dione (**2**, 578 mg, 2 mmol) in 10 mL of methanol was added to the reaction mixture and left stirred for 10 hours. The solvent was removed under vacuum and 20 mL of diethyl ether was added, the mixture was filtered, 15 mL of hexane was added to the mother liquor and set to crystallize. Red-brown crystals. (307 mg, 43%); m.p. 151 °C; Anal. Calcd for $C_{18}H_{18}N_2O_2S_2$: C, 60.31; H, 5.06; N, 7.81; S, 17.89. Found: C, 60.01; H, 4.95; N, 7.66; S, 17.44; ^{1}H NMR (CDCl$_3$, 400 MHz): δ 1.44 (s, 18H); ^{13}C{1H} NMR (CDCl$_3$, 100 MHz): δ 30.1, 38.1, 71.0, 111.1, 144.9, 146.3, 173.1, 183.7; IR (cm^{-1}): 2220, 1666, 1641, 1395, 1365, 1288, 1222, 1161, 1094, 1026, 982, 905, 762, 615, 526.

4,7-Di-*tert*-butyl-2-(3,5-di-*tert*-butyl-4-oxocyclohexa-2,5-dien-1-ylidene)benzo[*d*][1,3]dithiole-5,6-dione (**6c**) 60% suspension of NaH in mineral oil (136 mg, 3.4 mmol) was washed with dry diethyl ether (10 mL) and resulting solid was suspended in 20 mL of dry THF. To this suspension was added to 353 mg (1.7 mmol) of 2,6-di-*tert*-butylphenol in 5 mL of THF, and the mixture was allowed to react at room temperature for 10 hours. After that the solution turned yellow-green color. Solution of carbon disulfide (131 mg, 1.7 mmol) in 10 mL THF was added dropwise to phenoxide salt mixture at 0 °C and then stirred for 2 hours. 4,5-Dichloro-3,6-di-*tert*-butyl-*o*-quinone **2** (500 mg, 1.7 mmol) in 10 mL of THF was added in portions and stirred for 2 h. After that the solvent was removed under vacuum. The precipitate was dissolved in diethyl ether and then

red solution was filtered. The filtrate was evaporated under vacuum. The product was isolated after column chromatography in a mixture of hexane-toluene (4:1). Violet crystals. (389 mg, 46%); m.p. 229 °C (decomposes); Anal. Calcd for $C_{29}H_{38}O_3S_2$: C, 69.84; H, 7.68; S, 12.86. Found: C, 69.99; H, 7.57; S, 12.44; ^1H NMR ($CDCl_3$, 400 MHz): δ 1.34 (s, 18H), 1.49 (s, 18H), 7.15 (s, 2H); $^{13}C\{1H\}$ NMR ($CDCl_3$, 100 MHz): δ 29.4, 30.2, 35.6, 37.9, 123.0, 126.0, 141.1, 144.4, 147.8, 148.8, 183.3, 185.7; IR: 1648, 1629, 1605, 1377, 1364, 1331, 1294, 1253, 1221, 1100, 1085, 1029, 978, 891, 881, 838, 818, 766, 722, 674, 534.

4,7-Di-*tert*-butyl-2-(2,4-dioxopentan-3-ylidene)benzo[*d*][1,3]dithiole-5,6-dione (6d) and 8,8-diacetyl-2,5-di-*tert*-butyl-7-thiabicyclo[4.2.0]octa-1,5-diene-3,4-dione (7). A 60% suspension of NaH in mineral oil (160 mg, 4 mmol) was washed with dry diethyl ether (10 mL) and resulting solid was suspended in 20 mL of dry THF. This suspension was added dropwise to 200 mg (2 mmol) of acetylacetone in 10 mL THF at vigorous stirring and then stirred for 1 h. Then solution of carbon disulfide (152 mg, 2 mmol) in 10 mL of THF was added dropwise at 0 °C and stirred for 30 minutes. After that solution of 3,6-di-*tert*-butyl-4,5-dichlorocyclohex-3,5-diene-1,2-dione (**2**, 578 mg, 2 mmol) in 10 mL of THF was added to the reaction mixture and left for 10 hours. The solvent was removed under vacuum and 10 mL of acetone was added, the mixture was filtered and products **7** and **8** were separated by column chromatography in a mixture of hexane-acetone (10:1).

4,7-Di-*tert*-butyl-2-(2,4-dioxopentan-3-ylidene)benzo[*d*][1,3]dithiole-5,6-dione (6d) Red-brown crystals. (102 mg, 13%); m.p. 159 °C; Anal. Calcd for $C_{20}H_{24}O_4S_2$: C, 61.20; H, 6.16; S, 16.34. Found: C, 60.92; H, 5.92; s, 16.66; ^1H ($CDCl_3$, 400 MHz): δ 1.45 (s, 18H); 2.53 (s, 6H); $^{13}C\{1H\}$ NMR ($CDCl_3$, 100 MHz): δ 30.2, 30.6, 37.5, 128.4, 111.1, 143.0, 150.2, 160.9, 183.7, 195.4; IR (cm^{-1}): 1696, 1664, 1627, 1449, 1386, 1304, 1278, 1211, 1171, 1113, 1021, 998, 902, 816, 762, 606

8,8-Diacetyl-2,5-di-*tert*-butyl-7-thiabicyclo[4.2.0]octa-1,5-diene-3,4-dione (7) Red crystals. (56 mg, 8%); m.p. 165 °C; Anal. Calcd for $C_{19}H_{24}O_4S$: C, 65.49; H, 6.94; S, 9.20. Found: C, 65.33; H, 7.04; S, 9.80; ^1H NMR ($CDCl_3$; 400 MHz): δ 1.27 (s, 9H), 1.19 (s, 9H), 2.53 (s, 6H); $^{13}C\{1H\}$ NMR ($CDCl_3$, 100 MHz): δ 28.7, 29.0 26.7, 36.1, 36.6, 74.5, 138.7, 144.1, 148.4, 151.0, 160.9, 177.3, 180.8, 198.7; IR (cm^{-1}): 1708, 1687, 1665, 1648, 1598, 1535, 1482, 1358, 1294, 1208, 1174, 1069, 1023, 988, 949, 845, 824, 787, 770, 721, 647, 617, 565.

Compound 8. A 60% suspension of NaH in mineral oil (160 mg, 4 mmol) was washed with dry diethyl ether (10 mL) and resulting solid was suspended in 20 mL of dry THF. This suspension was added dropwise to 416 mg (2 mmol) of hexafluoroacetylacetone in 10 mL THF at vigorous stirring and then stirred for 1 h. Then solution of carbon disulfide (152 mg, 2 mmol) in 10 mL of THF was added dropwise at 0 °C and stirred for 30 minutes. After that solution of 3,6-di-*tert*-butyl-4,5-dichlorocyclohex-3,5-diene-1,2-dione (**2**, 578 mg, 2 mmol) in 10 mL of THF was added to the reaction mixture and reflux for 10 hours. Cooling of the reaction mixture to −18 °C resulted in deep red crystals suitable for X-ray study. (379 mg, 47%); m.p. 164 °C; Anal. Calcd for $C_{19}H_{19}$ Cl_2 F_6O_4Na: C, 43.95; H, 3.69. Found: C, 43.15; H, 3.70;

4,8-Di-*tert*-butyl-2,2,2-trimetoxy-2-[1,3]dithiole[4',5':4,5]benzo[1,2-*d*][1,3,2]dioxophosphol-6-thione (9) Trimethyl phosphite (124 mg, 1 mmol) was added dropwise to the solution 4,7-di-*tert*-butyl-2-thioxobenzo[*d*][1,3]dithiole-5,6-dione (326 mg, 1 mmol) in 10 mL of toluene and then stirred for 1 h. After that solvent was removed on rotary evaporator. Colorless powder was isolated after recrystallization from methanol. (440 mg, 98%); Anal. Calcd for $C_{18}H_{27}O_5PS_3$: C, 47.98; H, 6.04; P, 6.87; S, 21.35. Found: C, 47.57; H, 5.87; S, 20.90; ^1H NMR ($CDCl_3$, 400 MHz): δ 1.57(s, 18H), 3.53 (s, 9H); $^{13}C\{1H\}$ NMR ($CDCl_3$, 100 MHz): δ

30.86, 37.79, 128.2, 136.1, 143.2, 209.7; ^{31}P{1H} NMR ($CDCl_3$, 162 MHz): δ 11.6; IR (cm^{-1}): 1675, 1463, 1403, 1373, 1270, 1244, 1215, 1164, 1065, 1030, 980, 926, 904, 896, 836, 761, 723, 667, 635, 585, 534, 506, 468

4,7-Di-*tert*-butyl-5,6-dihydroxy-benzo[*d*][1,3]dithiole-2-thione. (10) Solution of $NaBH_4$ (0.116 mg, 3 mmol) in 15 mL of methanol was added dropwise to the solution 4,7-di-*tert*-butyl-2-thioxobenzo[*d*][1,3]dithiole-5,6-dione (326 mg, 1 mmol) in 15 mL methanol. The color of solution immediately turned from red to light-yellow. The mixture stirred for another 30 minutes, then solvent was removed on a rotary evaporator. Precipitate was washed with diethyl ether and solvent was removed on a rotary evaporator. Bright-yellow powder (308 mg, 94%); Anal. Calcd for $C_{15}H_{20}O_2S_3$: C, 54.84; H, 6.14; S, 29.28. Found: C, 55.12; H, 6.22; S, 29.55; ^{1}H NMR ($CDCl_3$, 400 MHz): δ 1.50(s, 18H); ^{13}C{1H} NMR ($CDCl_3$; 100 MHz): δ 31.0, 37.2, 121.7, 127.6, 154.0, 207.6; IR (cm^{-1}): 3500, 1705, 1615, 1543, 1415, 1392, 1379, 1366, 1266, 1198, 1162, 1047, 950, 855, 840, 816, 796, 744, 725,664, 603, 510

X-ray diffractometry data

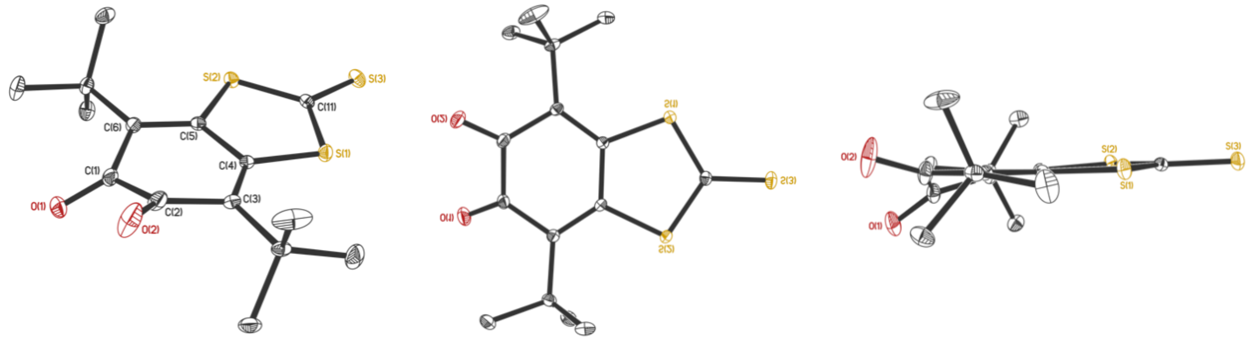

Figure S1. The molecular structure of **6a**. The thermal ellipsoids are given at 30% probability level. Hydrogen atoms are omitted for clarity.

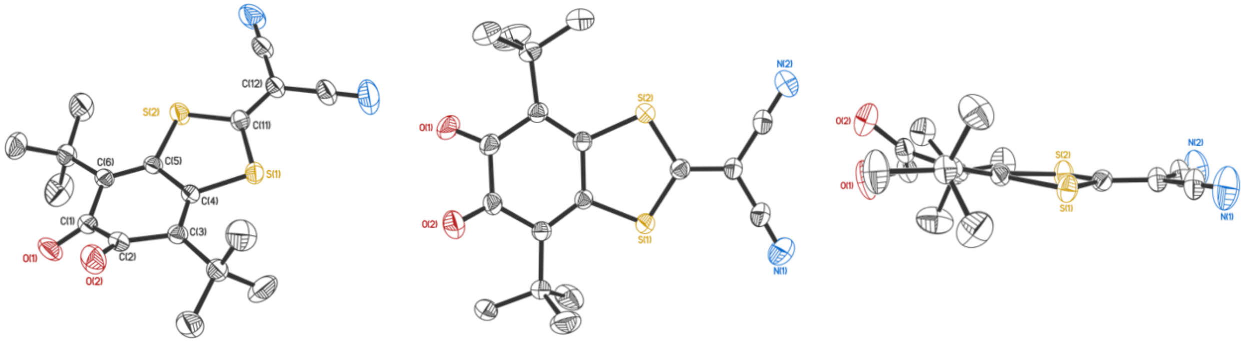

Figure S2. The molecular structure of **6b**. The thermal ellipsoids are given at 30% probability level. Hydrogen atoms are omitted for clarity.

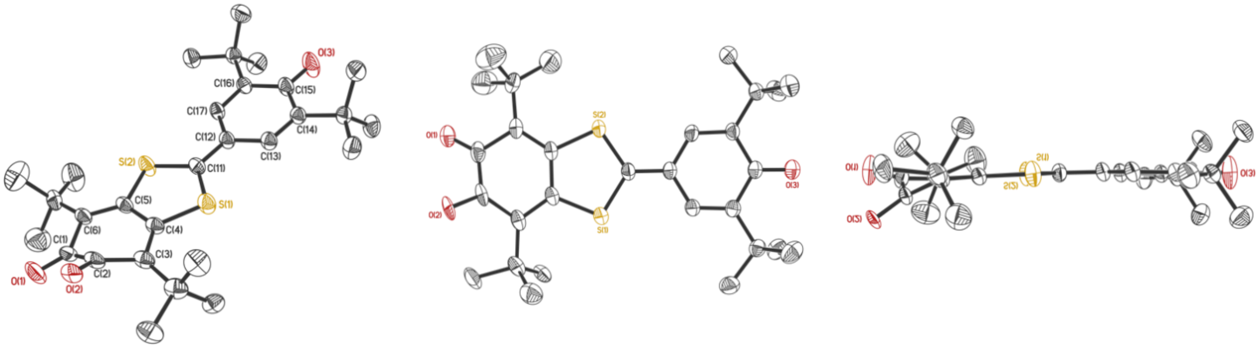

Figure S3. The molecular structure of **6c**. The thermal ellipsoids are given at 30% probability level. Hydrogen atoms are omitted for clarity.

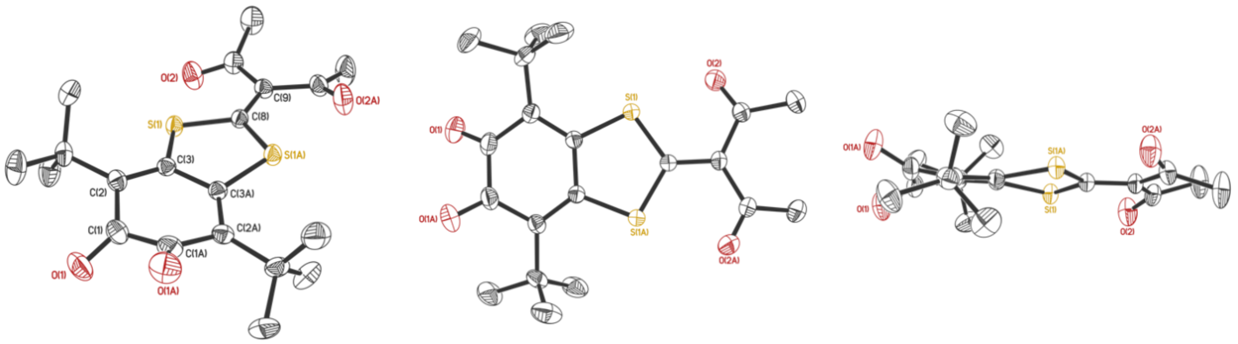

Figure S4. The molecular structure of **6d**. The thermal ellipsoids are given at 30% probability level. Hydrogen atoms are omitted for clarity.

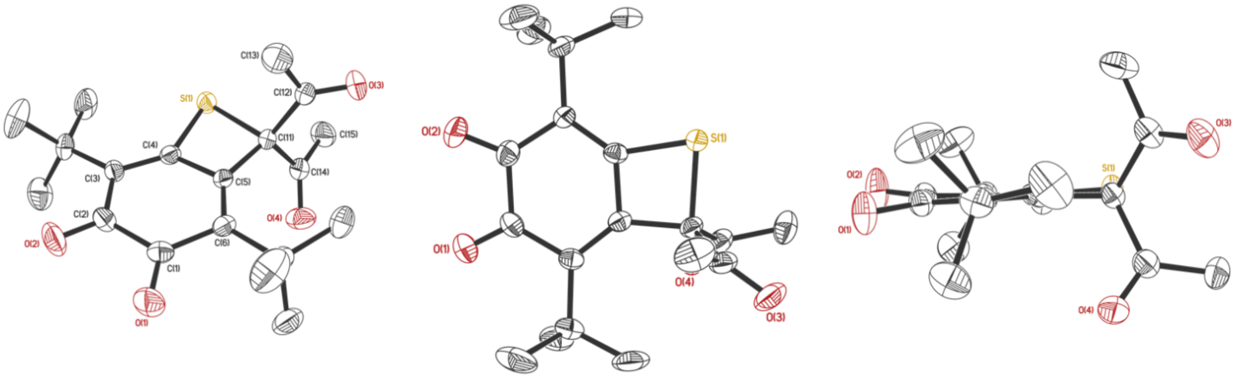

Figure S5. The molecular structure of **7**. The thermal ellipsoids are given at 30% probability level. Hydrogen atoms are omitted for clarity.

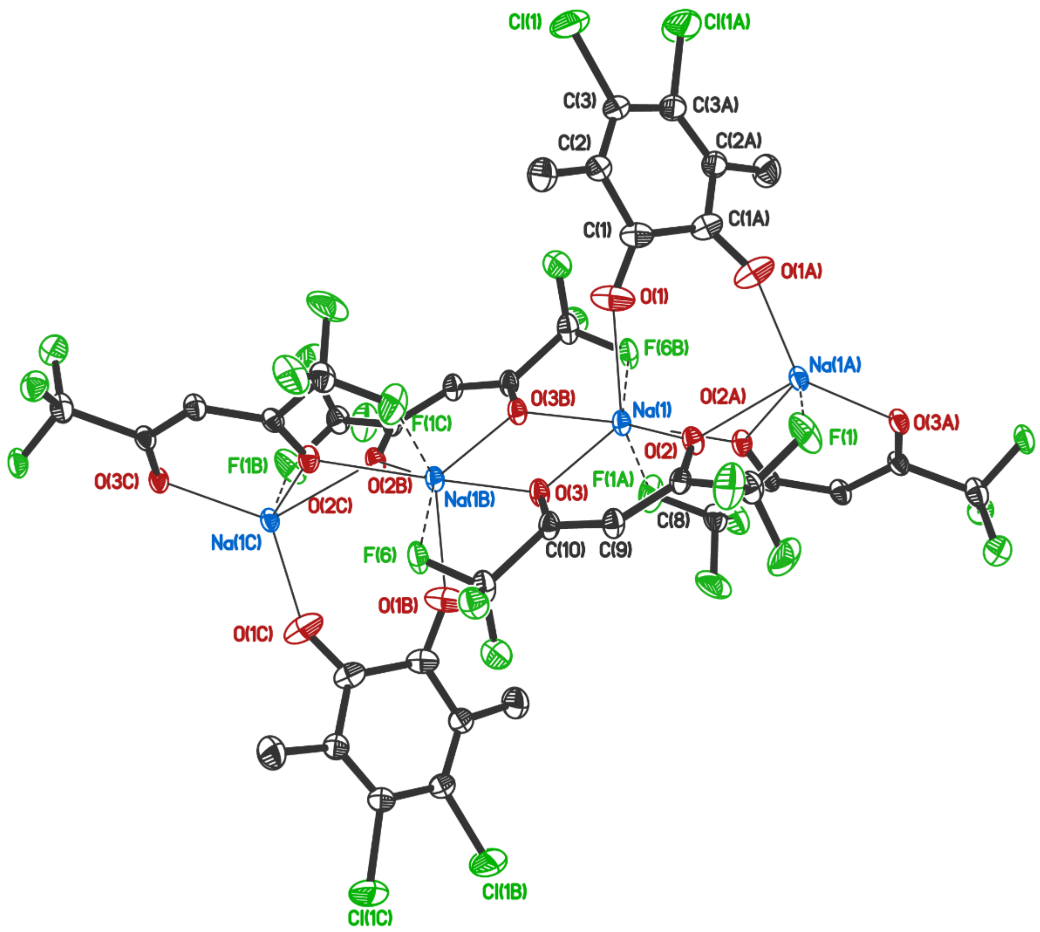

Figure S6. The fragment of crystal packing of **8**. The thermal ellipsoids are given at 10% probability level. Methyl groups of *tert*-butyl substituents and hydrogen atoms are omitted for clarity.

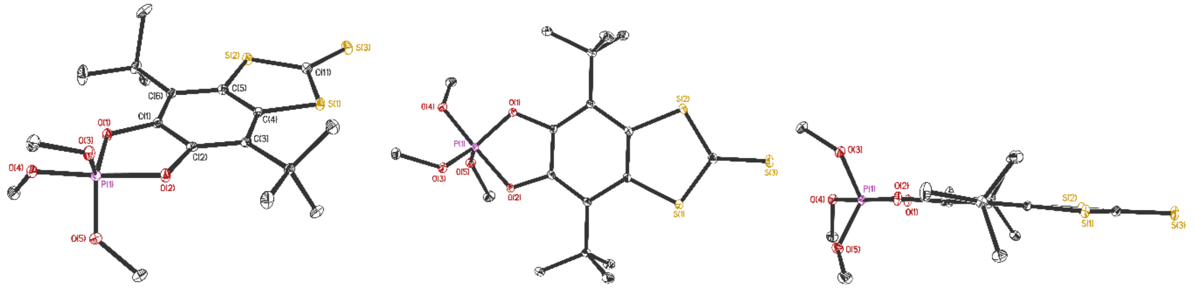

Figure S7. The molecular structure of **9**. The thermal ellipsoids are given at 30% probability level. Hydrogen atoms are omitted for clarity.

Table S1. Selected bond lengths (Å) and angles (deg) in **6a–d** and **7–9**.

	6a	6b	6c	6d	7	8	9
**Bond lengths**							
C=O	1.213(2)<br>1.214(2)	1.210(4)<br>1.204(4)	1.220(7)<br>1.224(6)	1.222(3)	1.206(4)<br>1.208(4)	1.209(7)	1.378(2)<br>1.347(2)
C(1)–C(2)	1.529(2)	1.530(5)	1.51(2)		1.560(5)		1.404(2)
C(1)–C(1A) {**6d**, **8**}				1.512(5)		1.49(2)	
C(1)–C(6)<br>C(2)–C(3)	1.473(2)<br>1.475(2)	1.478(4)<br>1.476(4)	1.480(6)<br>1.487(6)		1.492(5)<br>1.450(5)		1.385(2)<br>1.388(2)
C(1)–(C2) {**6d**, **8**}				1.461(3)		1.454(8)	
C(3)–C(4)<br>C(5)–C(6)	1.369(2)<br>1.359(2)	1.358(4)<br>1.356(4)	1.365(4)<br>1.366(4)		1.344(5)<br>1.341(5)		1.422(2)<br>1.424(2)
C(2)–C(3) {**6d**, **8**}				1.358(4)		1.335(9)	
C(4)–C(5)	1.497(2)	1.499(4)	1.488(5)		1.471(5)		1.407(2)
C(3)–C(3A) {**6d**, **8**}				1.487(4)		1.49(2)	
C(3)-Cl(1) {**8**}						1.710(6)	
C(4)–S(1)	1.761(2)	1.759(3)	1.744(3)	1.757(2)	1.754(4)		1.760(2)
C(5)–S(2)	1.760(2)	1.763(3)	1.748(3)	1.757(2)			1.763(2)
C(11)–S(1)	1.721(2)	1.713(3)	1.730(3)		1.866(3)		1.712(2)
C(11)–S(2)	1.720(2)	1.717(3)	1.722(3)				1.719(2)
C(8)–S(1) {**6d**}				1.736(2)			
C(8)–C(9) {**6d**}				1.378(4)			
C(8)-O(2) {**8**}						1.233(5)	
C(10)-O(3) {**8**}						1.243(5)	
C(11)–S(3)	1.633(2)						1.649(2)
C(11)–C(12)		1.370(4)	1.381(4)				
C(15)–O(3) {**6c**}			1.229(4)				
C(5)–C(11) {**7**}					1.531(5)		
C(11)–C(12) {**7**}					1.539(5)		
C(11)–C(14) {**7**}					1.537(5)		
P(1)–O(1)<br>P(1)–O(2)							1.644(2)<br>1.730(2)
P(1)–O(3)<br>P(1)–O(4)<br>P(1)–O(5)							1.581(2)<br>1.621(2)<br>1.586(2)
Na(1)-O(1)<br>Na(1)-O(2)<br>Na(1)-O(3)<br>Na(1)-O(2A)<br>Na(1)-O(3B)						2.324(5)<br>2.343(3)<br>2.334(3)<br>2.366(3)<br>2.358(3)	

Na(1)-F(1A)<br>Na(1)-F(6B)						2.93(2)<br>2.711(9)	
**Angles**							
C(4)–S(1)–C(11)	99.58(6)	97.5(2)	99.1(2)				98.86(7)
C(5)–S(2)–C(11)	99.08(6)	98.0(2)	99.2(2)				98.77(7)
C(3)–S(1)–C(8) {**6d**}				97.98(9)			
C(4)–S(1)–C(11) {**7**}					77.4(2)		

Table S2. Crystal data and structure refinement details for **6a–d** and **7–9**.

	**6a**	**6b**	**6c**	**6d**	**7**	**8**	**9**
Formula	$C_{15}H_{18}O_2S_3$	$C_{18}H_{18}N_2O_2S_2$	$C_{29}H_{38}O_3S_2$	$C_{20}H_{24}O_4S_2$	$C_{19}H_{24}O_4S$	$C_{24}H_{20}Cl_2F_{12}Na_2O_6$	$C_{18}H_{27}O_5PS_3$
M	326.47	358.46	498.71	392.51	348.44	749.28	450.54
T, K	100	RT	RT	RT	RT	RT	120
Crystal system	Orthorhombic	Monoclinic	Tetragonal	Monoclinic	Triclinic	Monoclinic	Triclinic
Space group	*Pbcn*	*C2/c*	*P–4*	*C2/c*	*P–1*	*C2/c*	*P–1*
a, Å	16.0608(5)	19.7539(10)	21.2299(3)	18.9124(6)	10.7397(5)	22.8517(13)	10.0514(3)
b, Å	9.0336(3)	8.8316(5)	21.2299(3)	12.4062(3)	11.5285(6)	11.8715(7)	11.4433(3)
c, Å	21.3669(6)	42.501(2)	6.12083(15)	8.6601(2)	15.2929(9)	13.6750(8)	11.4729(3)
α, deg	90	90	90	90	91.288(4)	90	110.139(2)
β, deg	90	101.516(5)	90	98.817(3)	97.502(5)	117.484(2)	112.187(3)
γ, deg	90	90	90	90	90.912(5)	90	102.860(2)
V, Å^3	3100.06(17)	7265.3(7)	2758.70(11)	2007.92(9)	1876.43(17)	3291.1(3)	1048.62(6)
Z	8	16	4	4	4	4	2
d_{calc}, g/cm^3	1.399	1.311	1.201	1.298	1.233	1.152	1.427
μ, mm^{-1}	0.476	0.305	0.220	0.287	0.191	0.327	0.456
F_{000}	1376	3008	1072	832	744	1504	476
Crystal dimensions, mm	0.48×0.35×0.18	0.38×0.24×0.12	0.41×0.17×0.16	0.52×0.27×0.26	0.46×0.24×0.15	0.65×0.25×0.08	0.83×0.30×0.09
θ range for data collection, deg	2.19–30.19	3.15–26.02	3.03–30.03	2.96–26.02	3.00–25.08	2.87–25.03	3.14–30.03
HKL indices	$-22 \le h \le 22$<br>$-12 \le k \le 12$<br>$-30 \le l \le 30$	$-24 \le h \le 23$<br>$-10 \le k \le 9$<br>$-52 \le l \le 48$	$-29 \le h \le 29$<br>$-28 \le k \le 29$<br>$-8 \le l \le 8$	$-23 \le h \le 23$<br>$-15 \le k \le 15$<br>$-10 \le l \le 10$	$-12 \le h \le 12$<br>$-13 \le k \le 13$<br>$-18 \le l \le 18$	$-27 \le h \le 26$<br>$-14 \le k \le 13$<br>$-16 \le l \le 16$	$-14 \le h \le 14$<br>$-16 \le k \le 16$<br>$-16 \le l \le 16$
Reflns. collected	42096	15235	53100	15014	23401	9414	20323
Reflns. unique	4586	7112	7947	1984	23401	2883	6113
R_{int}	0.0352	0.0280	0.0500	0.0325	0.0581	0.0259	0.0329
Data / restraints / parameters	4586 / 0 / 187	7112 / 604 / 544	7947 / 553 / 419	1984 / 0 / 123	23401 / 476 / 516	2883 / 299 / 272	6113 / 60 / 268
$S(F^2)$	1.023	1.026	1.020	1.066	1.008	1.058	1.043
R_1/wR_2	0.0332 / 0.0836	0.0599 / 0.1423	0.0543 / 0.1358	0.0432 / 0.1186	0.0630 / 0.1425	0.0996 / 0.2451	0.0329 / 0.0764

($I > 2\sigma(I)$)							
R_1/wR_2 (all data)	0.0408 / 0.0884	0.0998 / 0.1664	0.0888 / 0.1556	0.0512 / 0.1259	0.1285 / 0.1563	0.1187 / 0.2606	0.0426 / 0.0803
Largest diff. peak and hole, e/Å^3	0.63 / –0.42	0.51 / –0.32	0.31 / –0.19	0.50 / –0.20	0.34 / –0.28	0.56 / –0.38	0.51 / –0.40

Table S3. Description of samples preparation for X-ray diffractometry study

Compound	Description
**6a**	By slow cooling of acetone-diethyl ether mixture (1:3 volume). Red-brown rectangular shaped crystals
**6b**	Dried mixture of products were dissolved in 20 ml of diethyl ether and then layered with 15 ml of hexane. Red-brown crystals.
**6c**	The product was dissolved in diethyl ether and then layered with a half-volume of hexane. Violet crystals.
**6d**	By slow evaporation of CH_2Cl_2/heptane (1:1) mixture at rt. Red-brown crystals.
**7**	By slow evaporation of CH_2Cl_2/heptane (1:1) mixture at rt. Red crystals.
**8**	By cooling of THF solution up to -18°C. Deep red crystals.
**9**	By slow evaporation of methanol solution. Colorless semitransparent crystals.

EPR spectroscopy data and EPR spectra

Table S4 Parameters of the isotropic EPR spectra of metal complexes with *o*-quinones **6a–d** and **7** at 293 K

quinone		Metallofragment (Mf), solvent			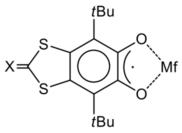		
		$^{7}Li/^{6}Li$, THF	Tl, THF	K; THF	$Mn(CO)_4$; toluene	H; toluene	$Mn(CO)_3Ph_3P$; toluene
**6a**	g	2.0055	2.0007	2.0053	2.0052	—	2.0045
	a_{Mf},(G)	0.50/ 0.19	61.82	—	7.30	—	11.20
	a_{P},(G)	—	—	—	—	—	35.14
**6b**	g	2.0041	1.9996	2.0043	2.0052	—	—
	a_{N},(G) 2xCN	0.17	—	0.18	0.22	—	—
	a_{Mf},(G)	0.47/ 0.18	66.14	—	7.80	—	—
**6c**	g	2.0044	1.9996	2.0044	2.0036	2.0048	2.0024
	a_{Mf},(G)	0.50/ 0.19	59.17	—	6.02	1.16	9.34
	a_{H},(G) 2xH	0.21	—	0.18	0.44	0.50	0.34
	a_{P},(G)	—	—	—	—	—	31.15
**6d**	g	2.0040	1.9990	2.0048	2.0030	2.0045	2.0024
	a_{Mf},(G)	0.52/ 0.20	60.00	—	7.10	1.18	11.10
	a_{H},(G) 2xCH_3	0.28	—	0.31	0.25	0.25	0.18
	a_{P},(G)	—	—	—	—	—	34.28
**7**	g	2.0045	1.9980	2.0046	2.0033	—	2.0029
	a_{Mf},(G)	0.58/ 0.23	57.50	—	6.80	—	9.60
	a_{H},(G) 2xCH_3	0.15	—	0.16	—	—	—
	a_{P},(G)	—	—	—	—	—	34.30

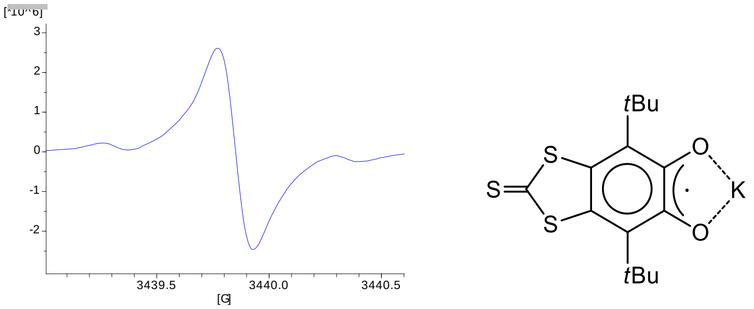

Figure S8. EPR spectrum of potassium semiquinonate with **6a** in THF solution, 293 K.

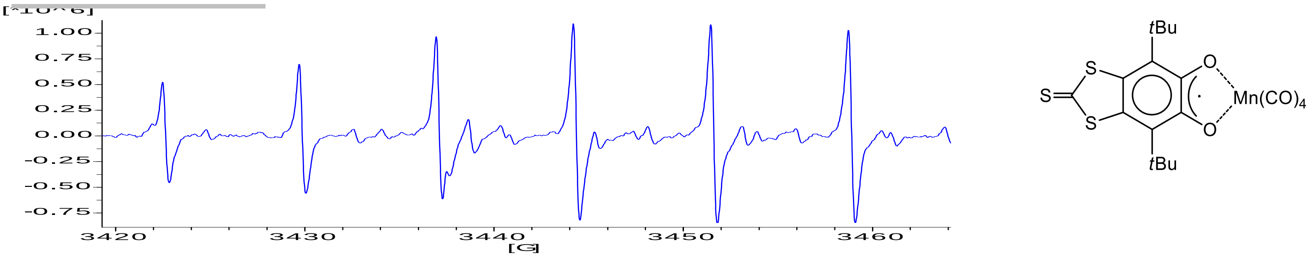

Figure S9. EPR spectrum of $Mn(CO)_4$ semiquinonate with **6a** in toluene solution, 293 K.

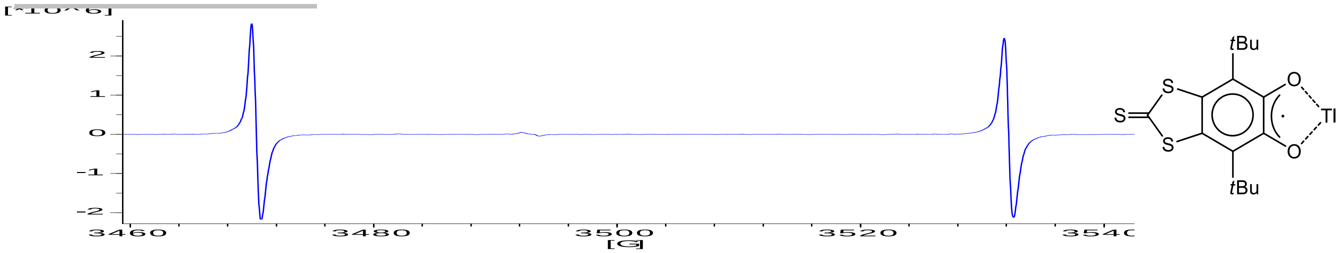

Figure S10. EPR spectrum of thallium semiquinonate with **6a** in THF solution, 293 K.

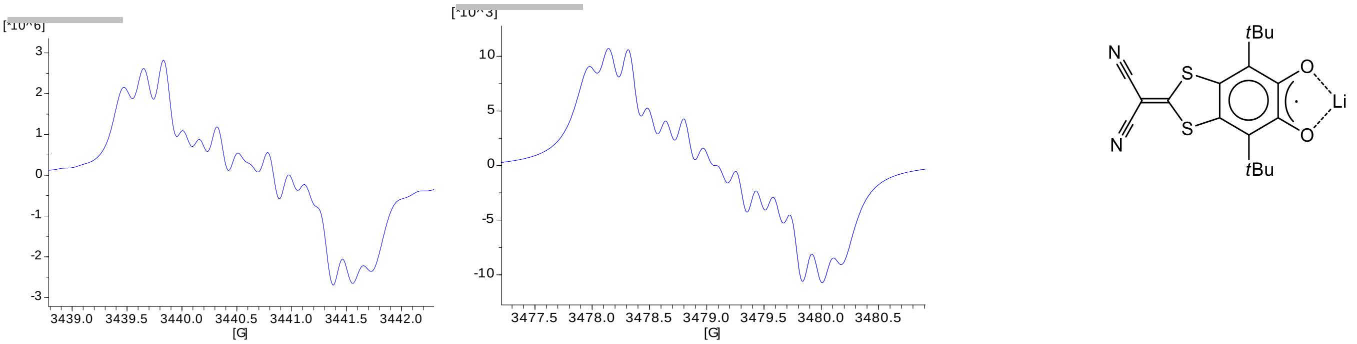

Figure S11. EPR spectrum of lithium semiquinonate with **6b** in THF solution, 293 K (left); simulated spectrum (right).

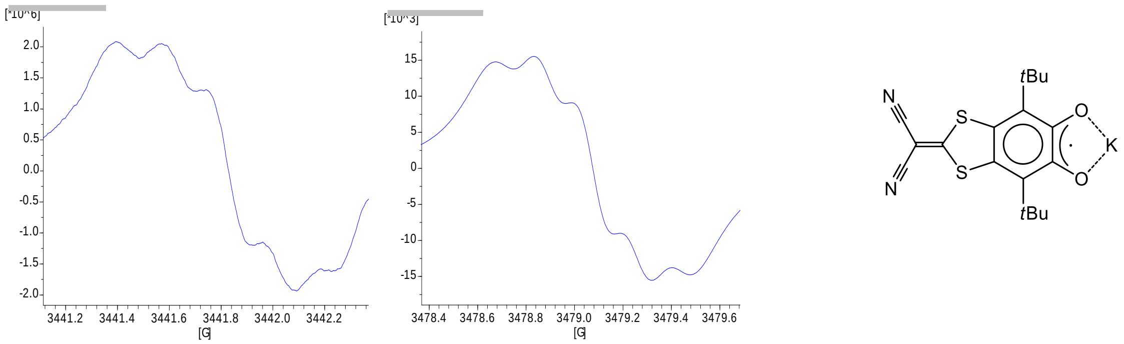

Figure S12. EPR spectrum of potassium semiquinonate with **6b** in THF solution, 293 K (left); simulated spectrum (right).

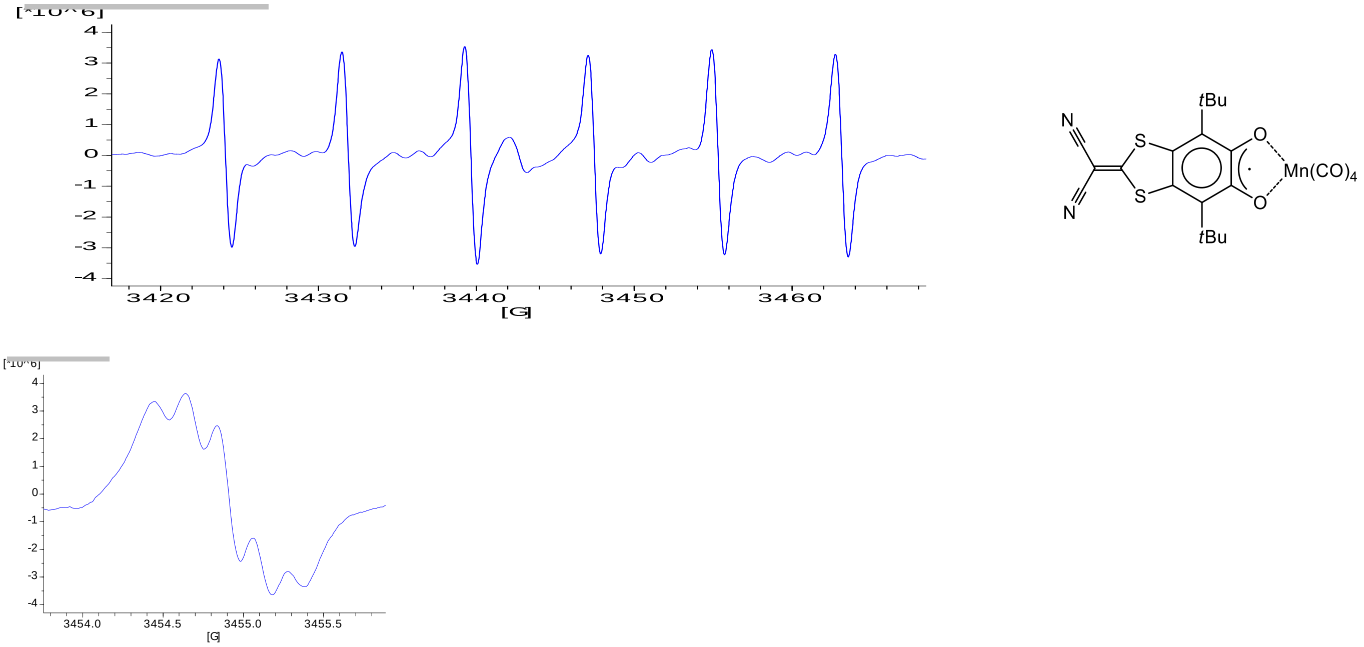

Figure S13. EPR spectrum of $Mn(CO)_4$ semiquinonate with **6b** in toluene solution, 293 K (top); resolved signal for 5th component of manganese sextet in diluted toluene solution (bottom)

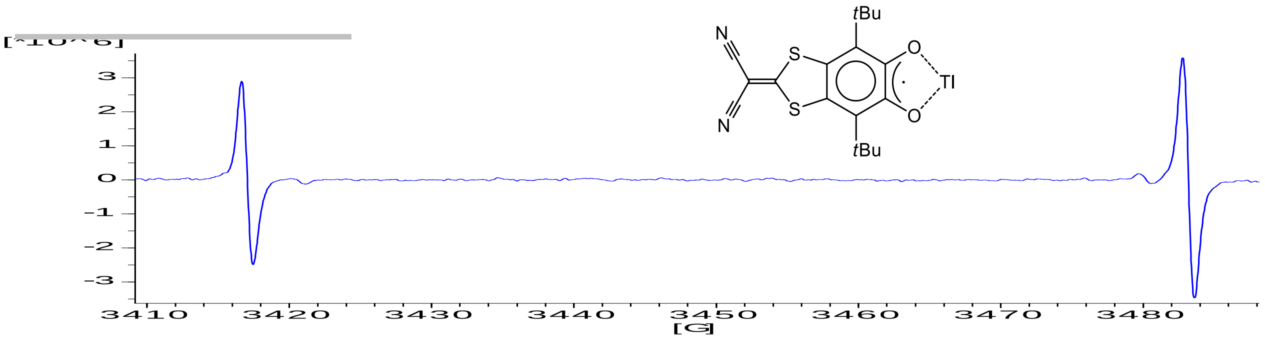

Figure S14. EPR spectrum of thallium semiquinonate with **6b** in THF solution, 293 K.

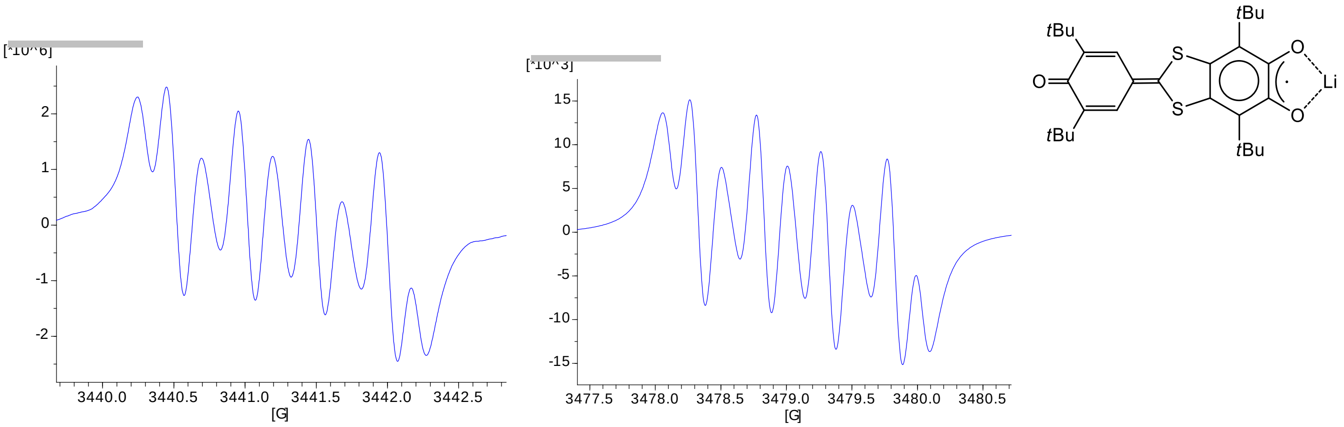

Figure S15. EPR spectrum of lithium semiquinonate with **6c** in THF solution, 293 K (left); simulated spectrum (right).

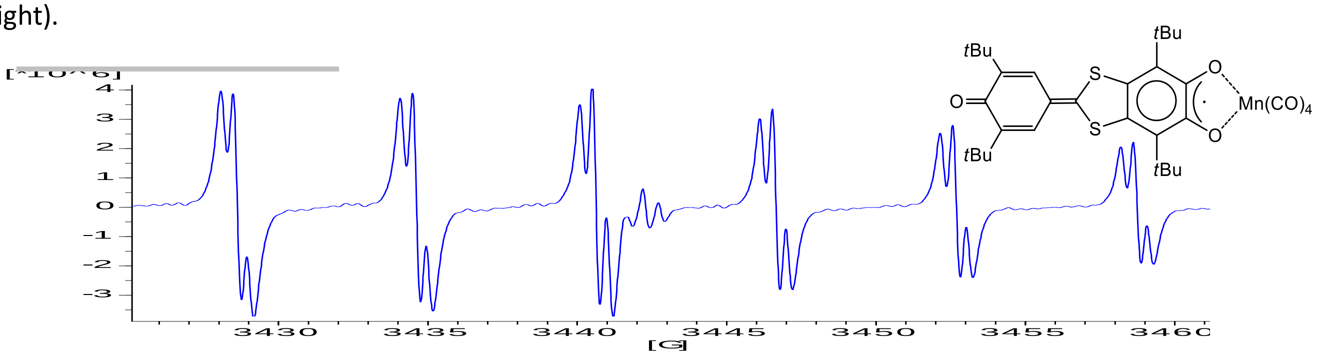

Figure S16. EPR spectrum of $Mn(CO)_4$ semiquinonate with **6c** in toluene solution, 293 K.

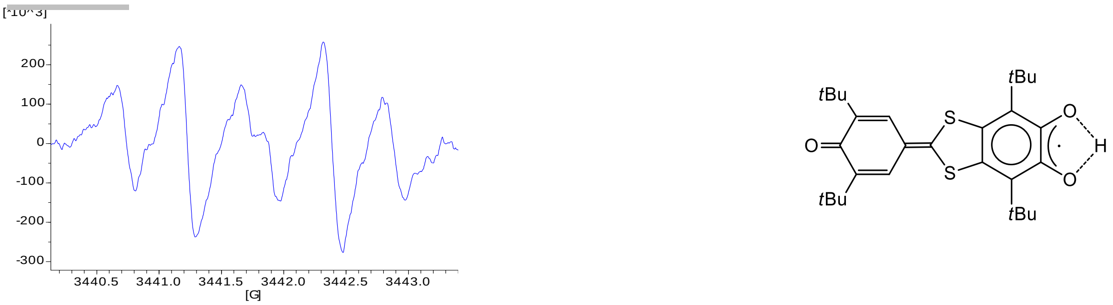

Figure S17. EPR spectrum of protonated semiquinone with **6c** in toluene solution, 293 K.

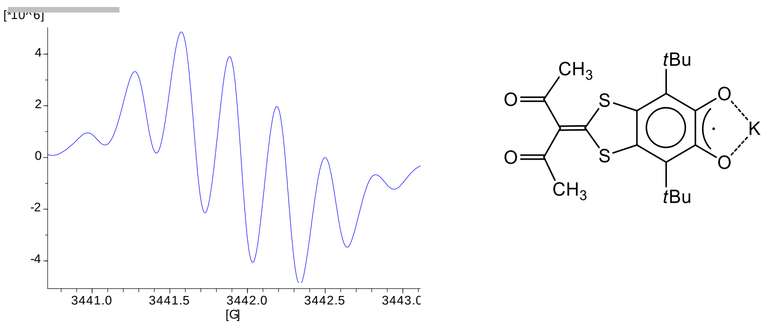

Figure S18. EPR spectrum of potassium semiquinonate with **6d** in THF solution, 293 K.

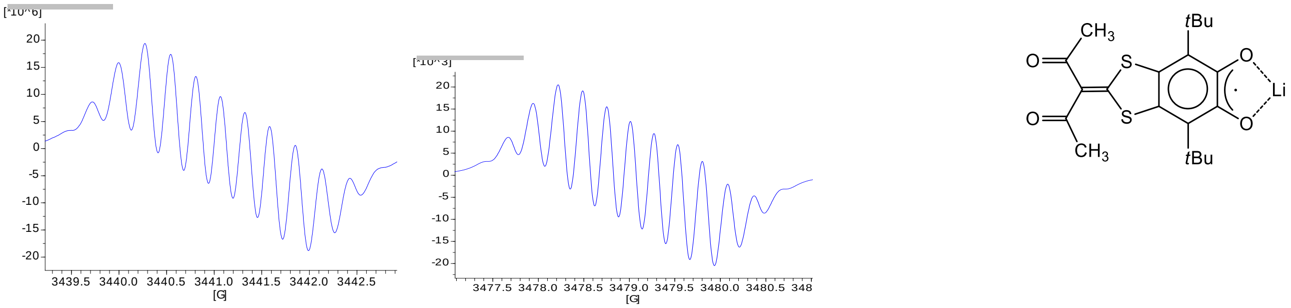

Figure S19. EPR spectrum of lithium semiquinonate with **6d** in THF solution, 293 K (left); simulated spectrum (right).

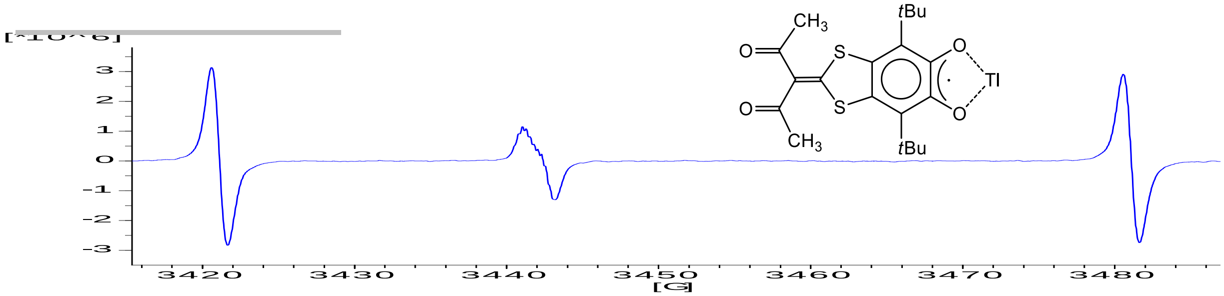

Figure S20. EPR spectrum of thallium semiquinonate with **6d** in THF solution, 293 K.

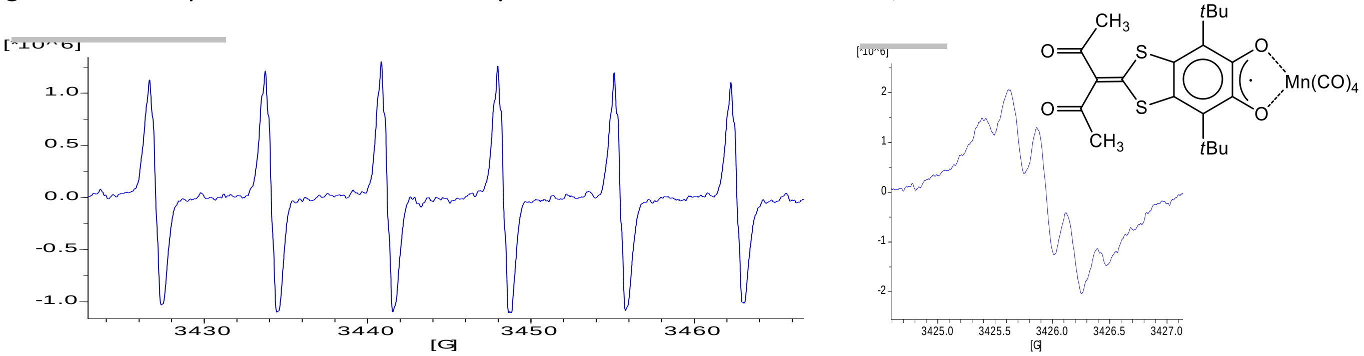

Figure S21. EPR spectrum of $Mn(CO)_4$ semiquinonate with **6d** in toluene solution, 293 K (left); resolved signal for low field component of manganese sextet in diluted toluene solution (right).

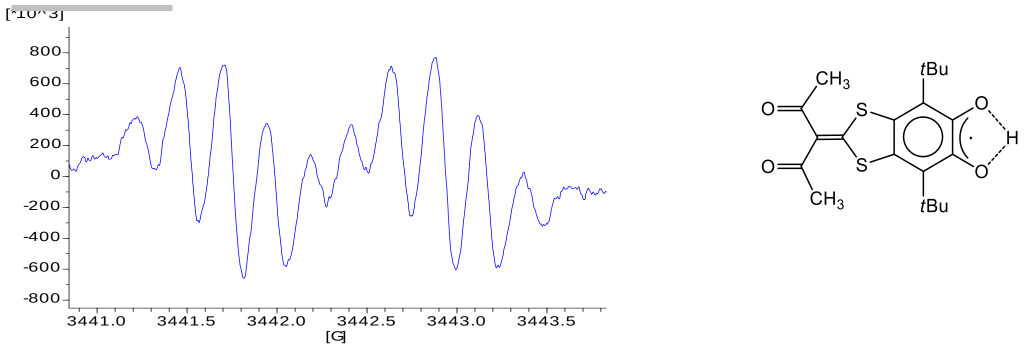

Figure S22. EPR spectrum of protonated semiquinone with **6d** in toluene solution, 293 K.

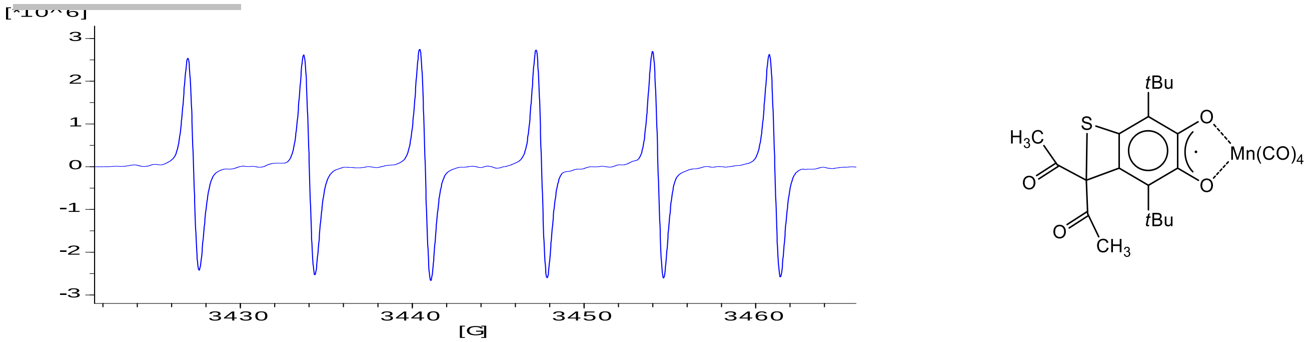

Figure S23. EPR spectrum of $Mn(CO)_4$ semiquinonate with **7** in toluene solution, 293 K.

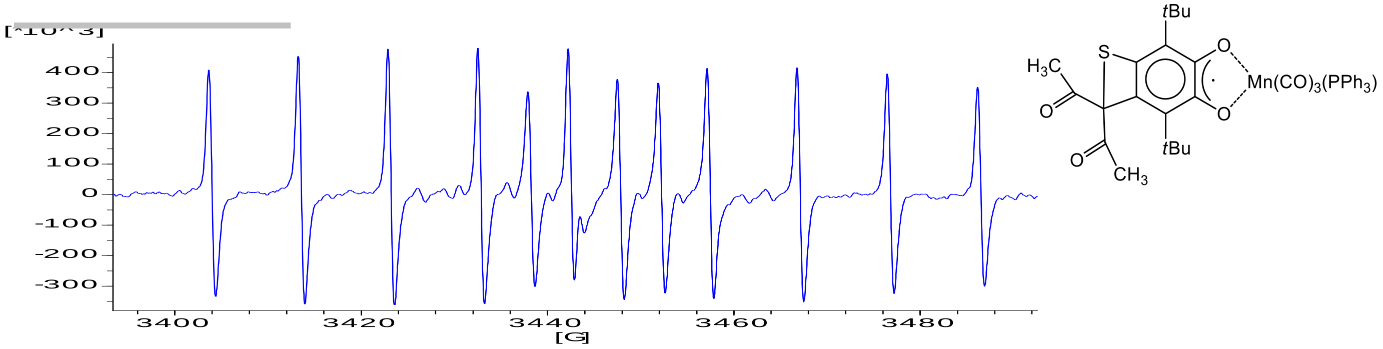

Figure S24. EPR spectrum of $Mn(CO)_3(PPh_3)$ semiquinonate with **7** in toluene solution, 293 K.

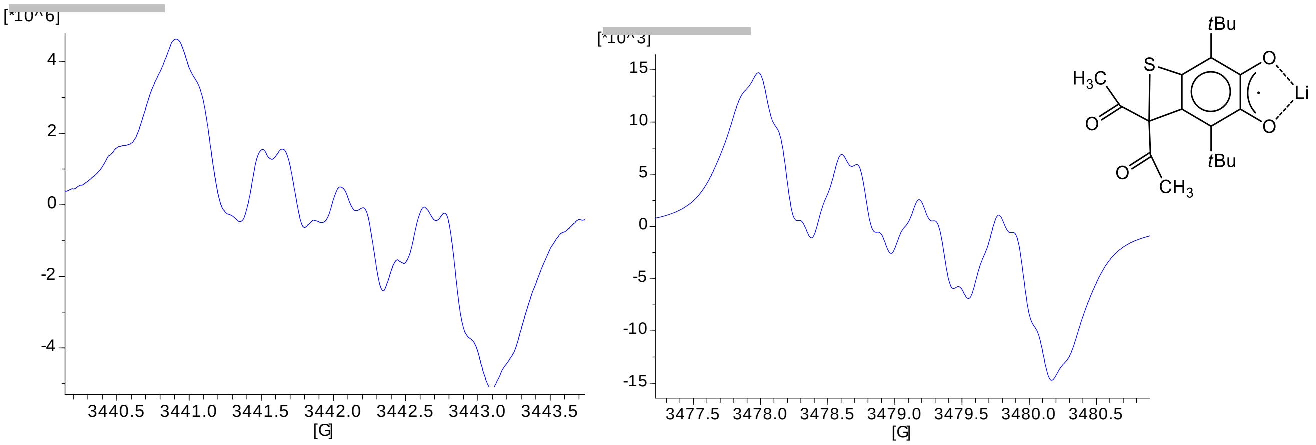

Figure S25. EPR spectrum of lithium semiquinonate with **7** in THF solution, 293 K (left); simulated spectrum (right).

ELectrochemistry data

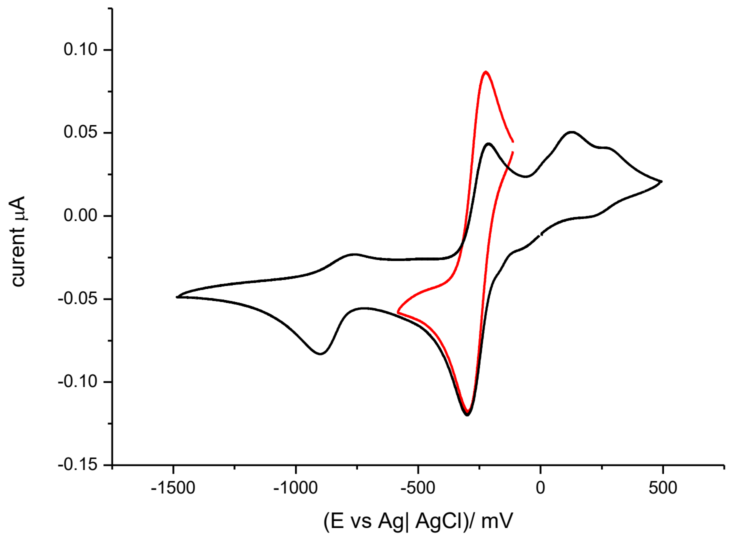

Figure S26. Cyclic voltammogram for compound **6a** (2×10^{-3} M), measured in CH_3CN B = 0.1 V/s, 0.1 M NBu_4ClO_4,. Ag/AgCl/KCl (sat.)).

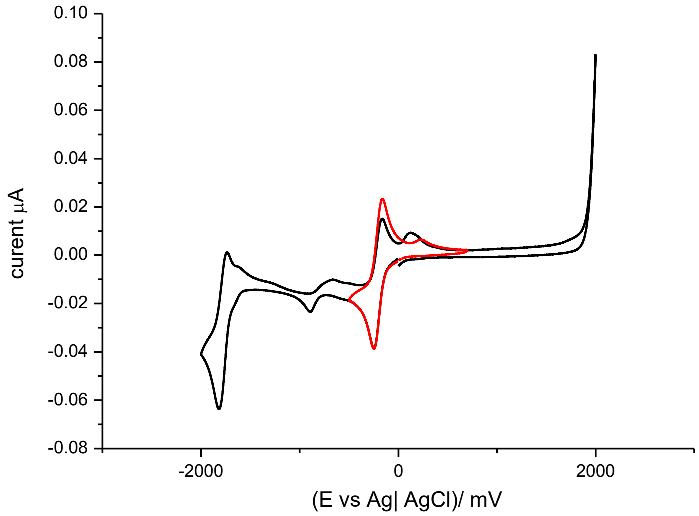

Figure S27. Cyclic voltammogram for compound **6b** (2×10^{-3} M), measured in CH_3CN B = 0.1 V/s, 0.1 M NBu_4ClO_4,. Ag/AgCl/KCl (sat.)).

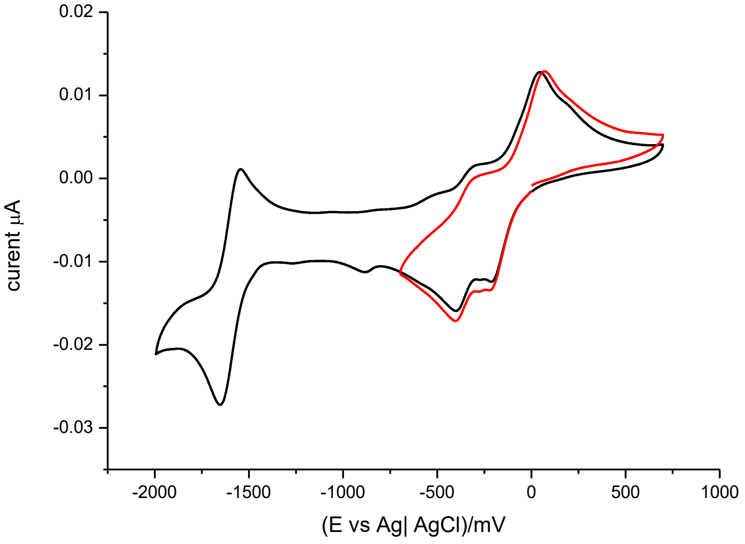

Figure S28. Cyclic voltammogram for compound **6c** (2×10^{-3} M), measured in CH_3CN B = 0.1 V/s, 0.1 M NBu_4ClO_4,. Ag/AgCl/KCl (sat.)).

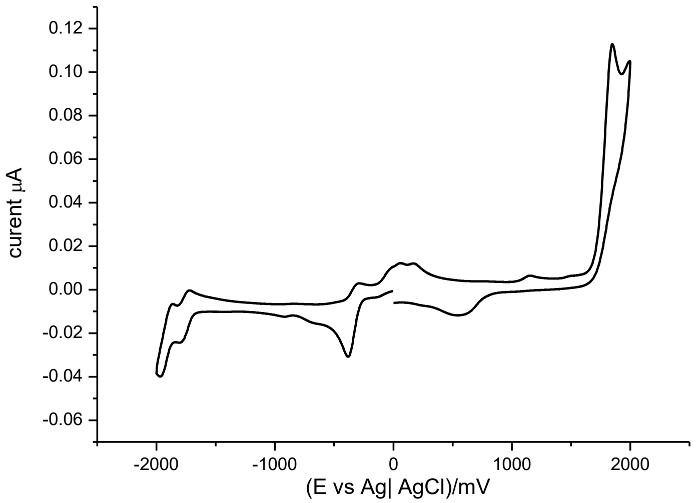

Figure S29. Cyclic voltammogram for compound **6d** (2×10^{-3} M), measured in CH_3CN B = 0.1 V/s, 0.1 M NBu_4ClO_4,. Ag/AgCl/KCl (sat.)).

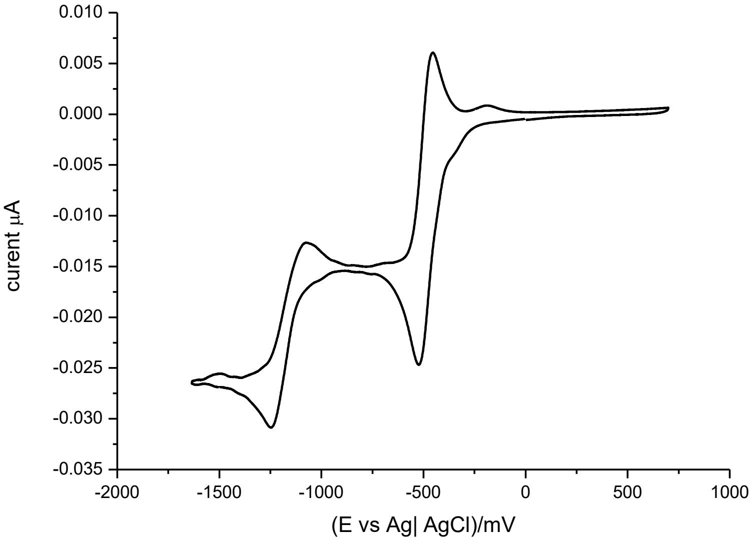

Figure S30. Cyclic voltammogram for compound **7** (2×10^{-3} M), measured in CH_3CN B = 0.1 V/s, 0.1 M NBu_4ClO_4,. Ag/AgCl/KCl (sat.)).

UV–vis spectra

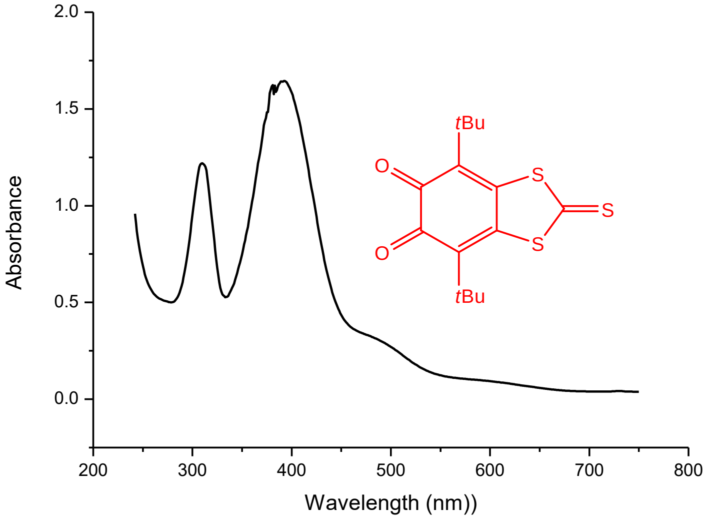

Figure S31. UV–vis spectrum of o-quinone **6a** in THF.

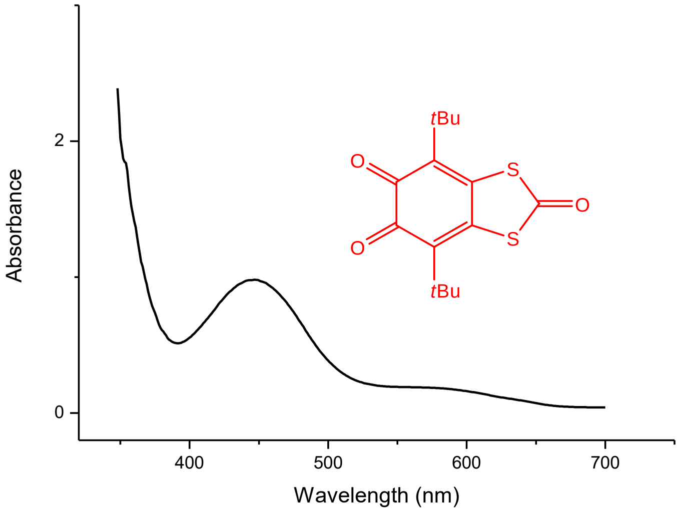

Figure S32. UV–vis spectrum of o-quinone **3a** in THF.

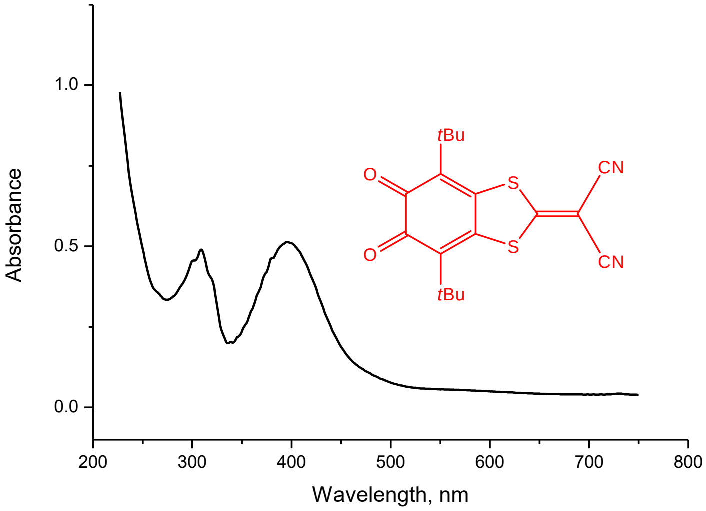

Figure S33. UV–vis spectrum of o-quinone **6b** in THF.

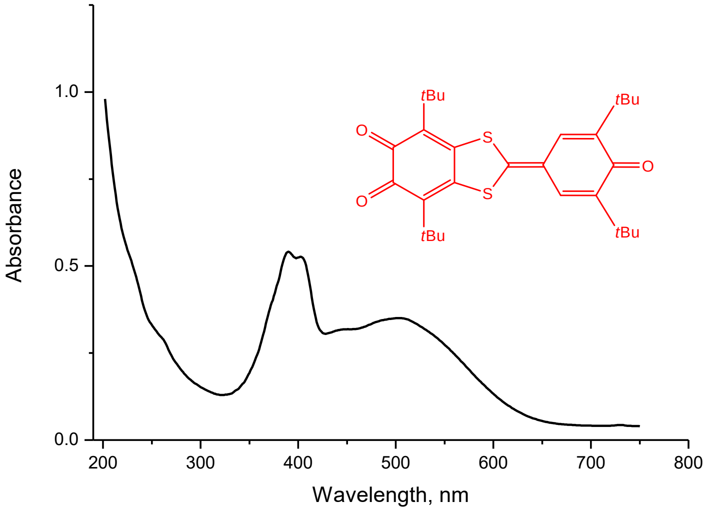

Figure S34. UV–vis spectrum of o-quinone **6c** in THF.

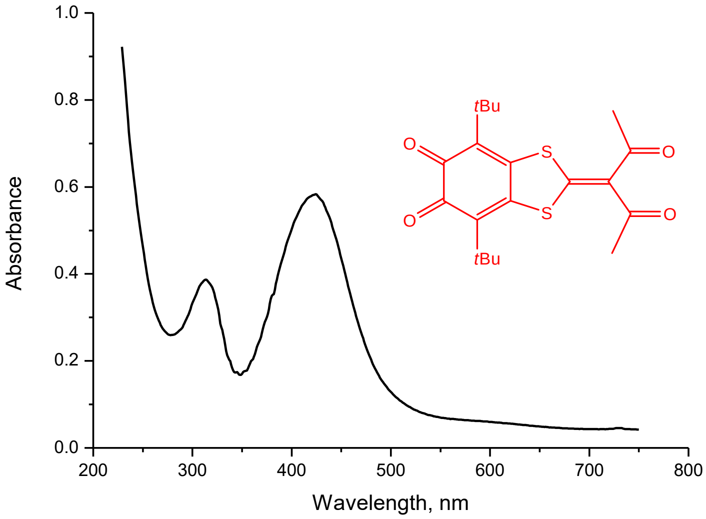

Figure S35. UV–vis spectrum of o-quinone **6d** in THF.

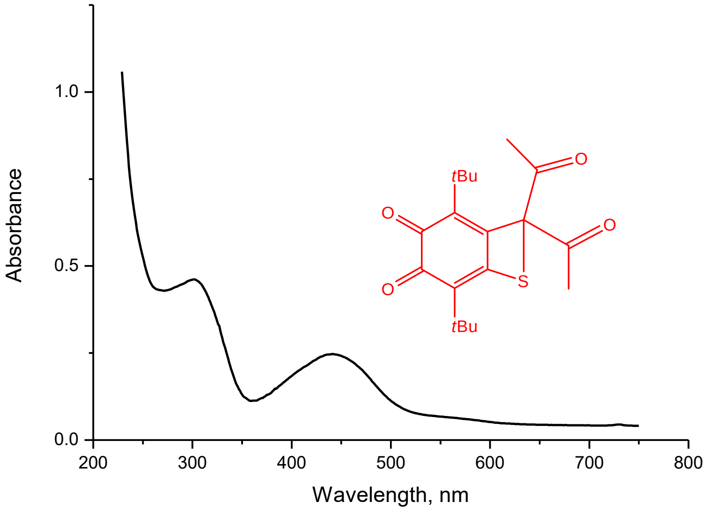

Figure S36. UV–vis spectrum of o-quinone **7** in THF.

NMR spectra

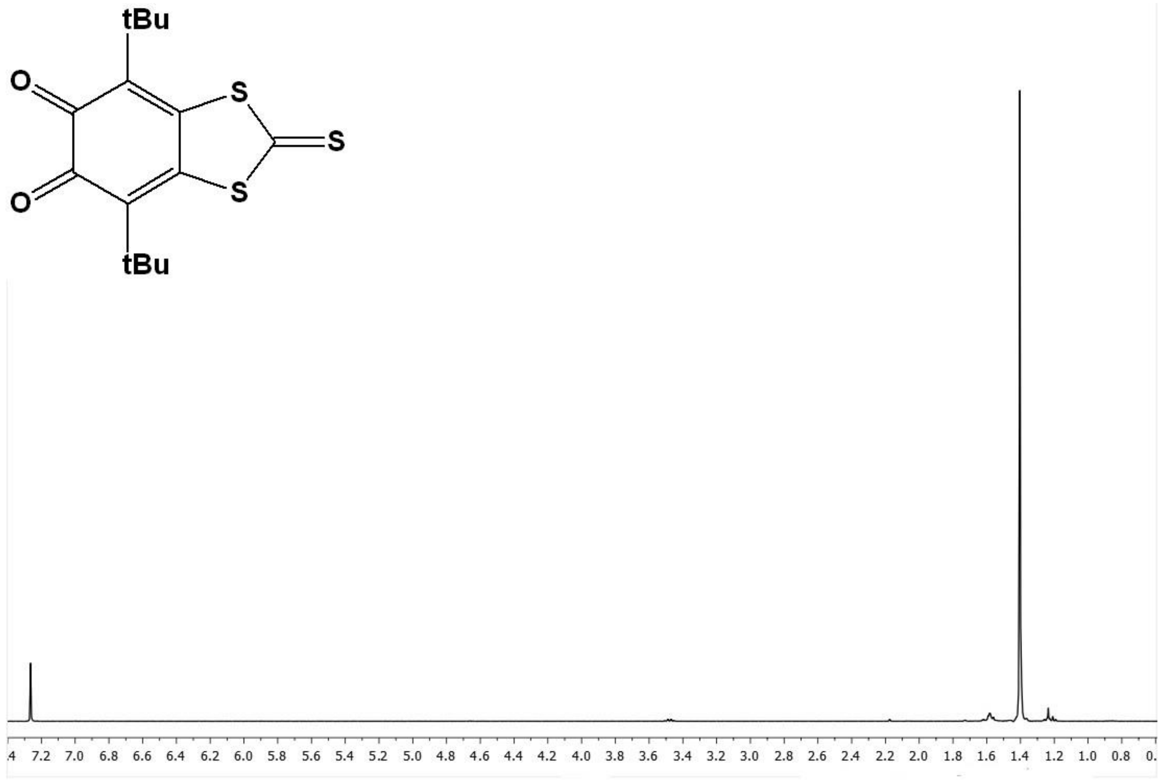

Figure S37. ^{1}H NMR spectrum of quinone **6a** in $CDCl_3$

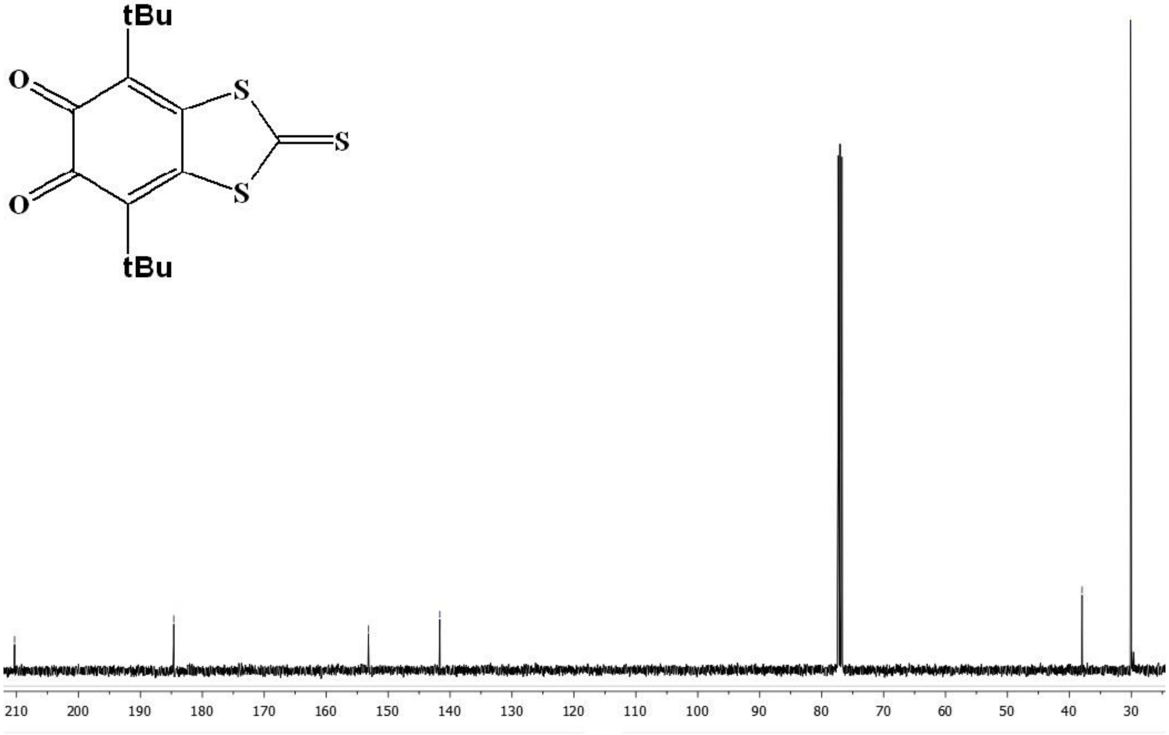

Figure S38. ^{13}C NMR spectrum of quinone **6a** in $CDCl_3$

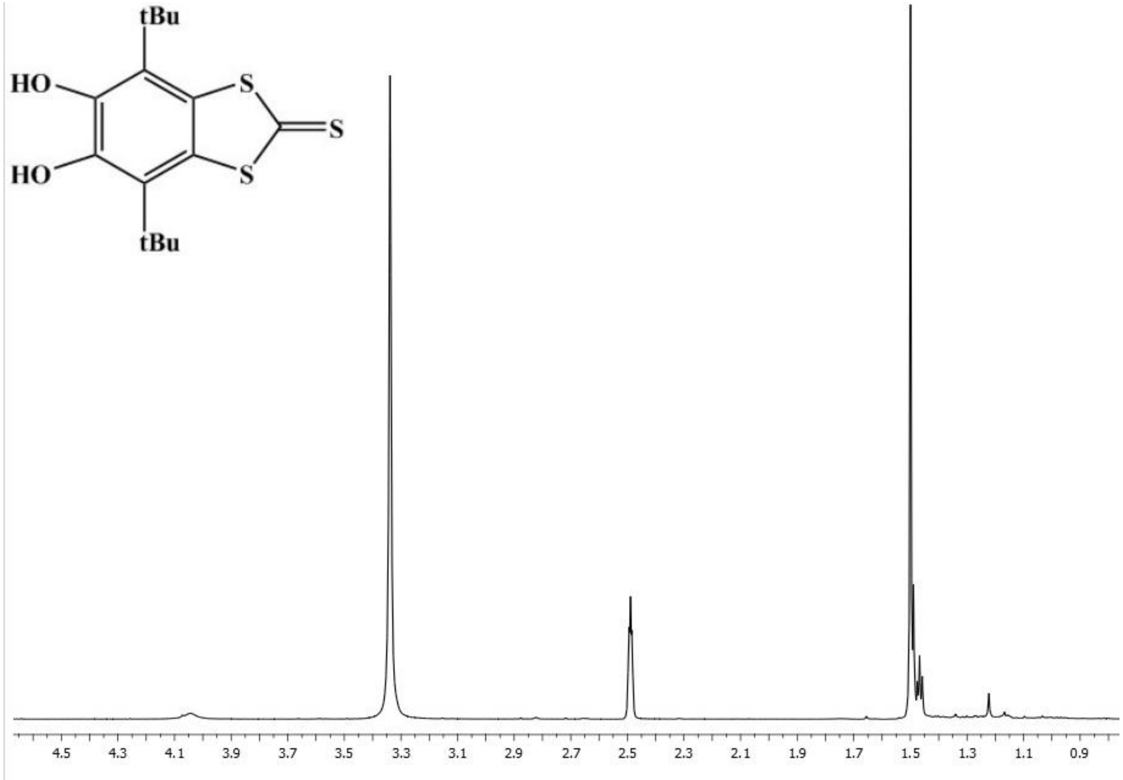

Figure S39. ^{1}H NMR spectrum of catechol **10** in DMSO-d_6

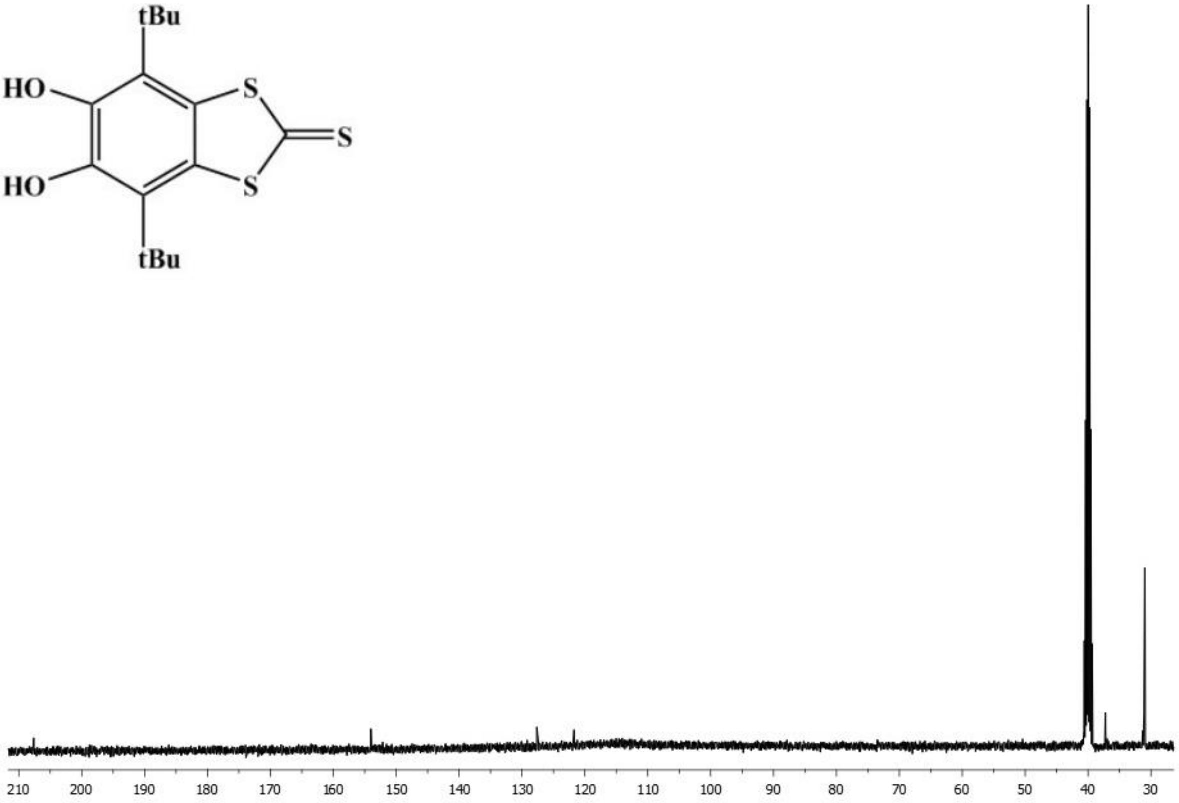

Figure S40. ^{13}C NMR spectrum of catechol **10** in DMSO-d_6

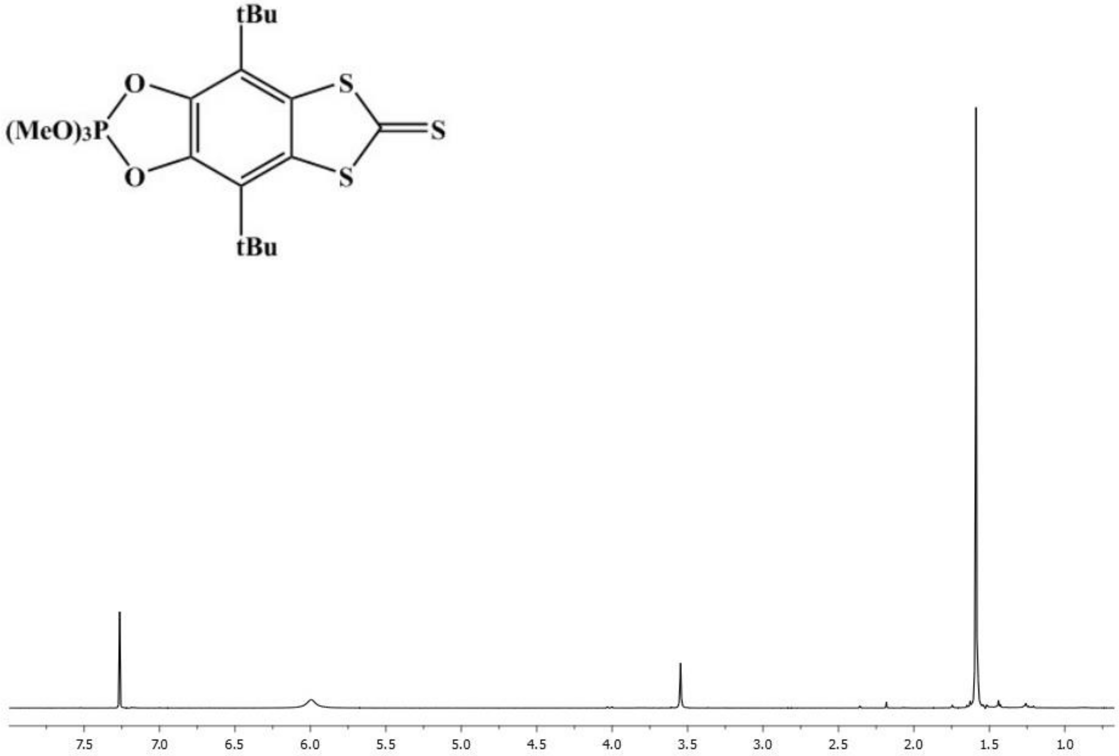

Figure S41. ^{1}H NMR spectrum of compound **9** in $CDCl_3$

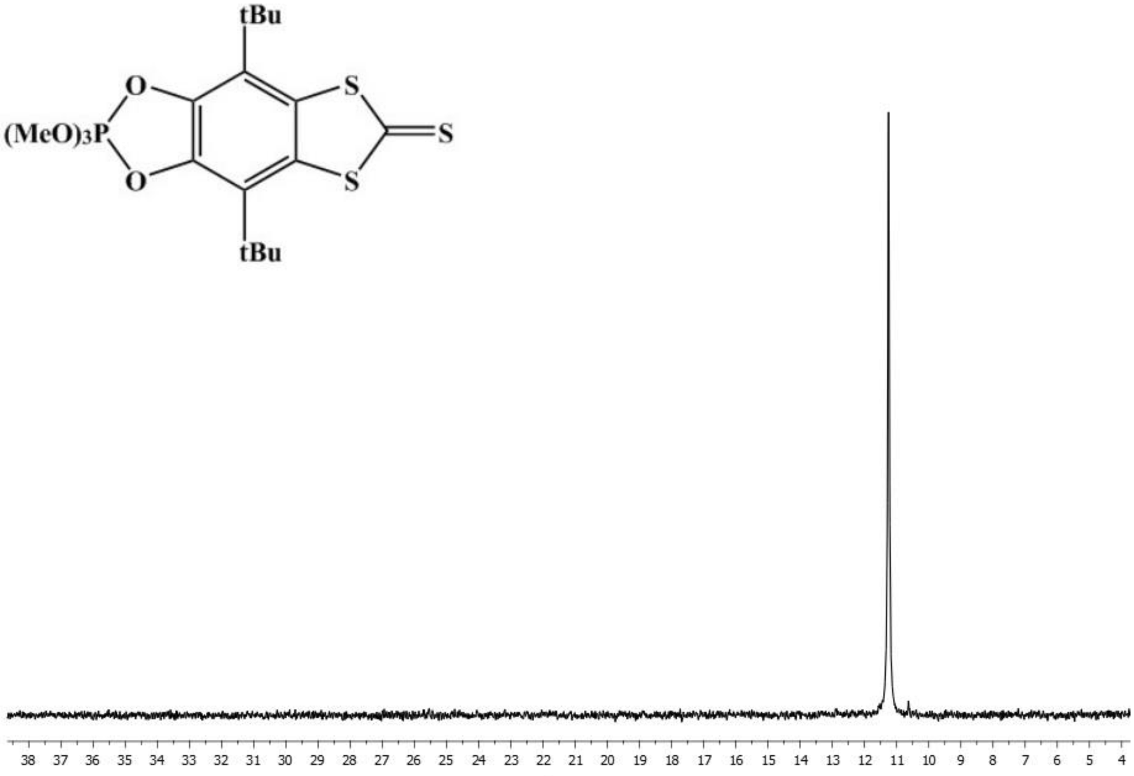

Figure S42. ^{31}P NMR spectrum of compound **9** in $CDCl_3$

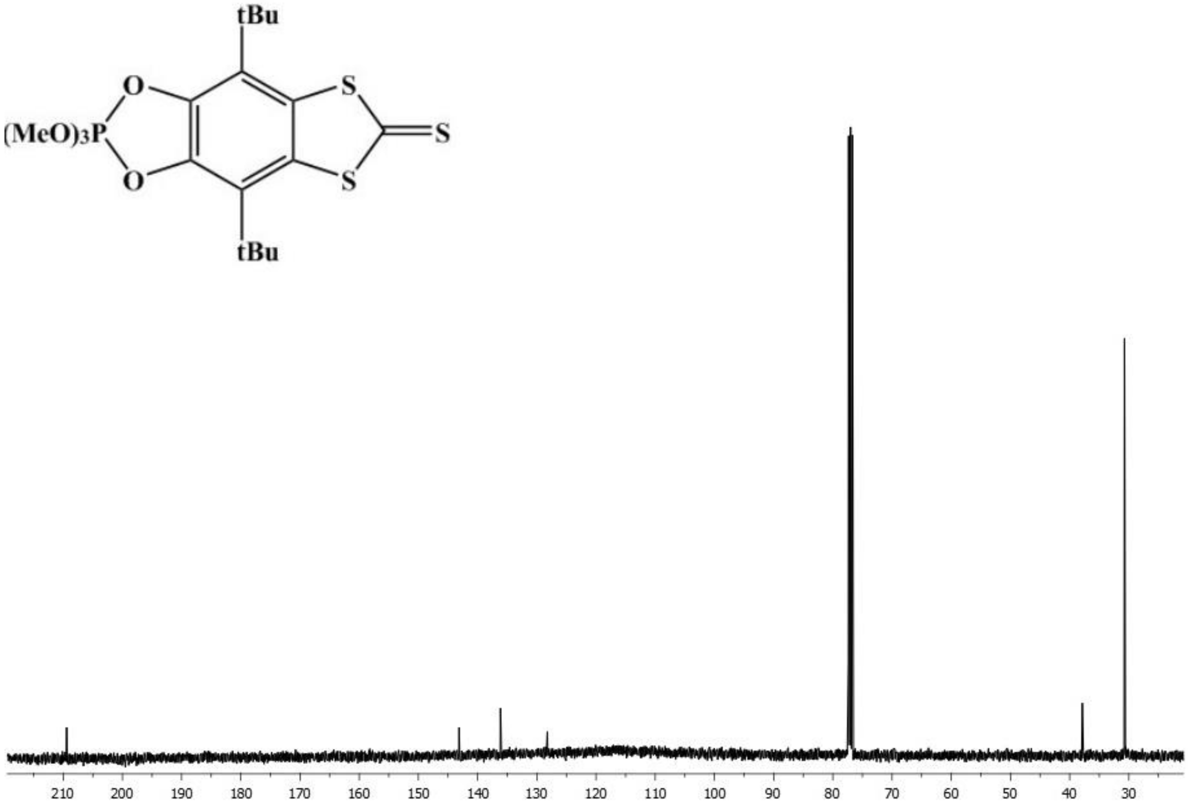

Figure S43. ^{13}C NMR spectrum of compound **9** in $CDCl_3$

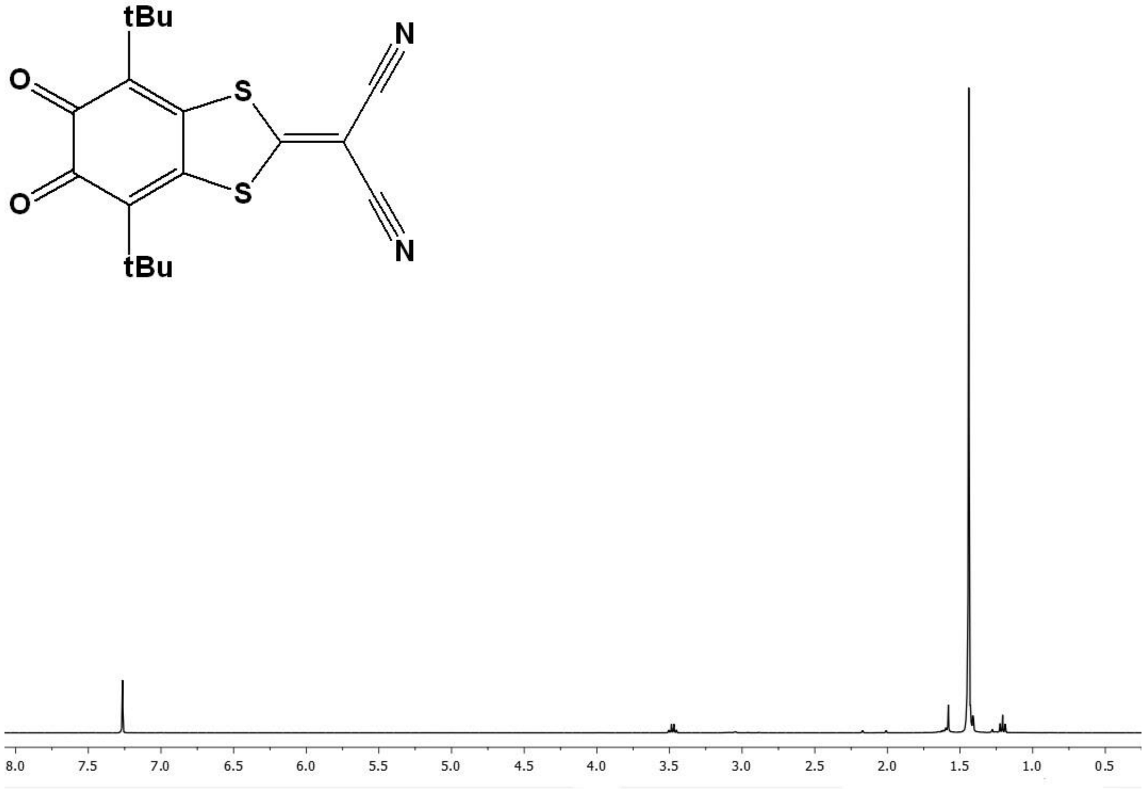

Figure S44. ^{1}H NMR spectrum of quinone **6b** in $CDCl_3$

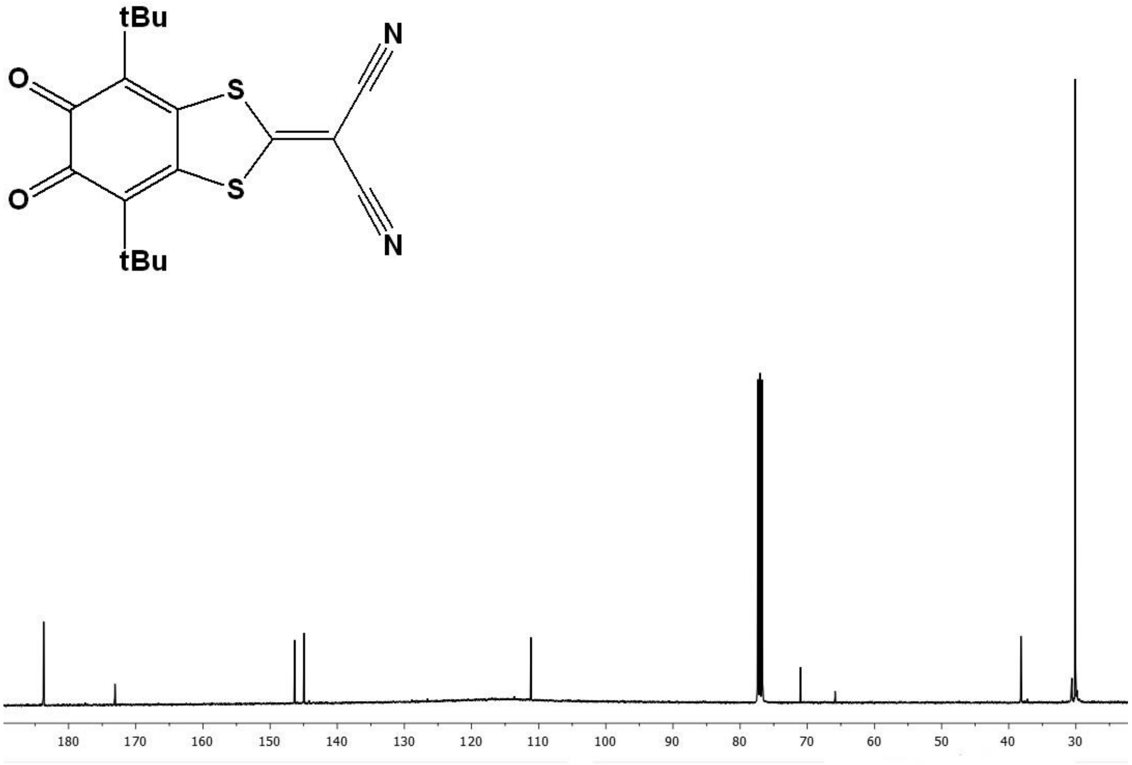

Figure S45. ^{13}C NMR spectrum of quinone **6b** in $CDCl_3$

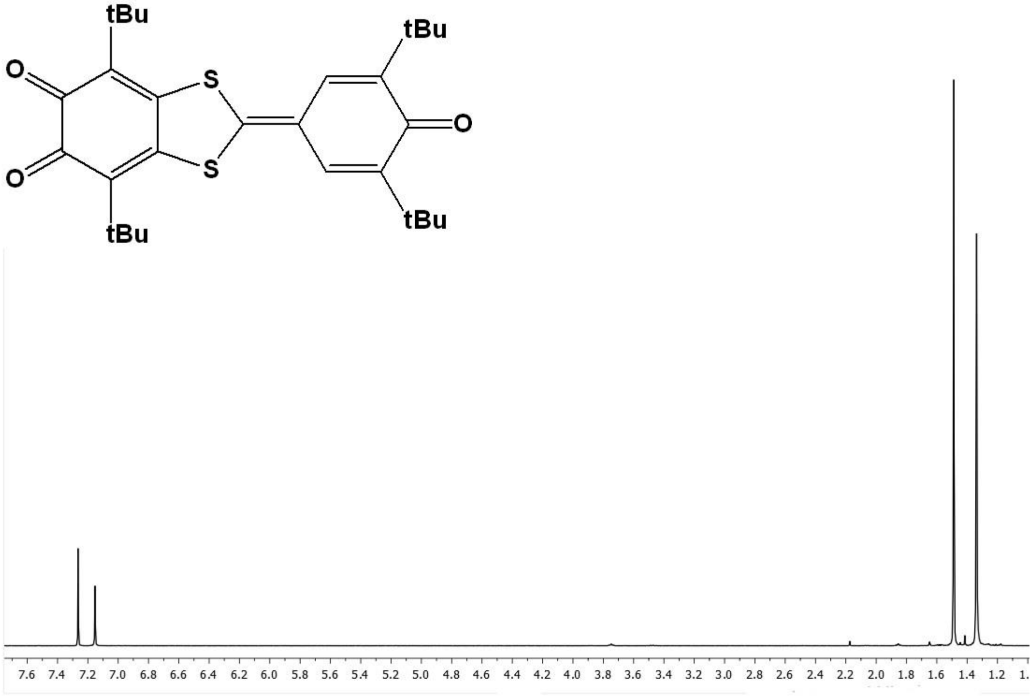

Figure S46. ^{1}H NMR spectrum of quinone **6c** in $CDCl_3$

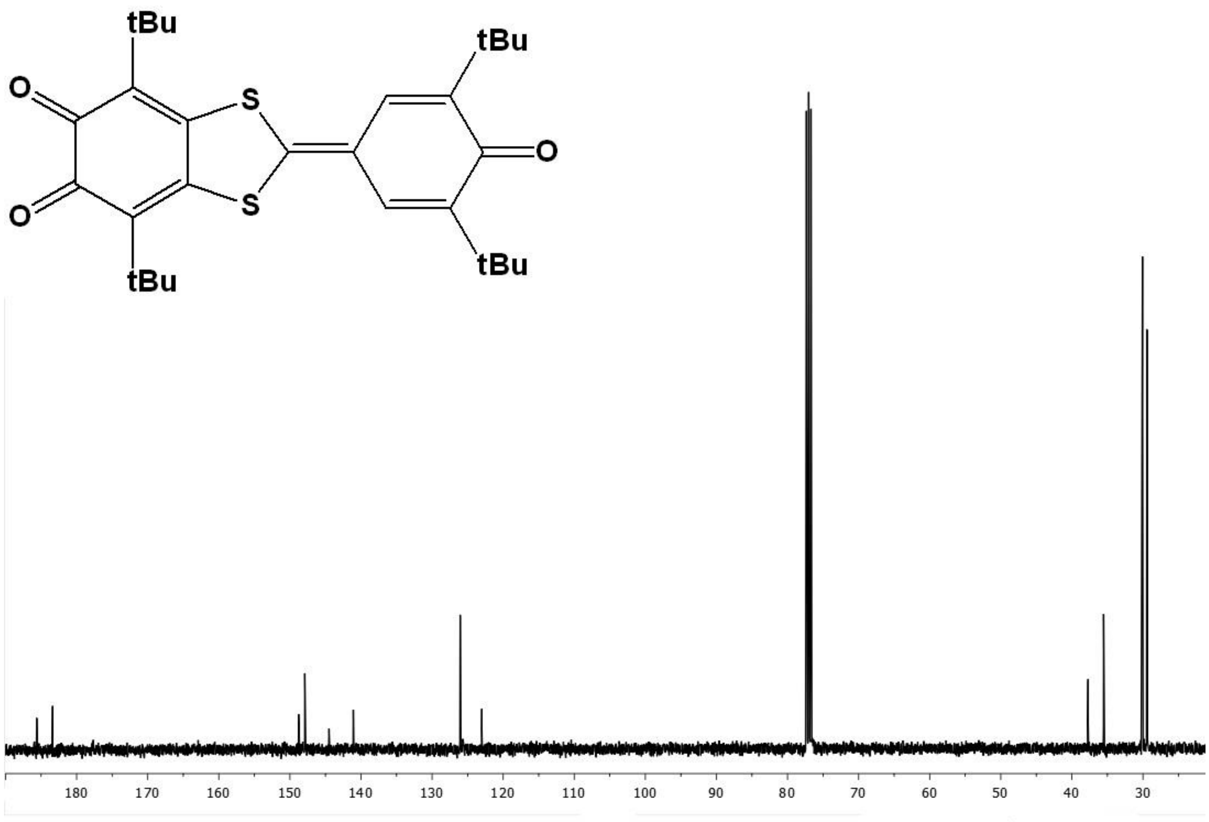

Figure S47. ^{13}C NMR spectrum of quinone **6c** in $CDCl_3$

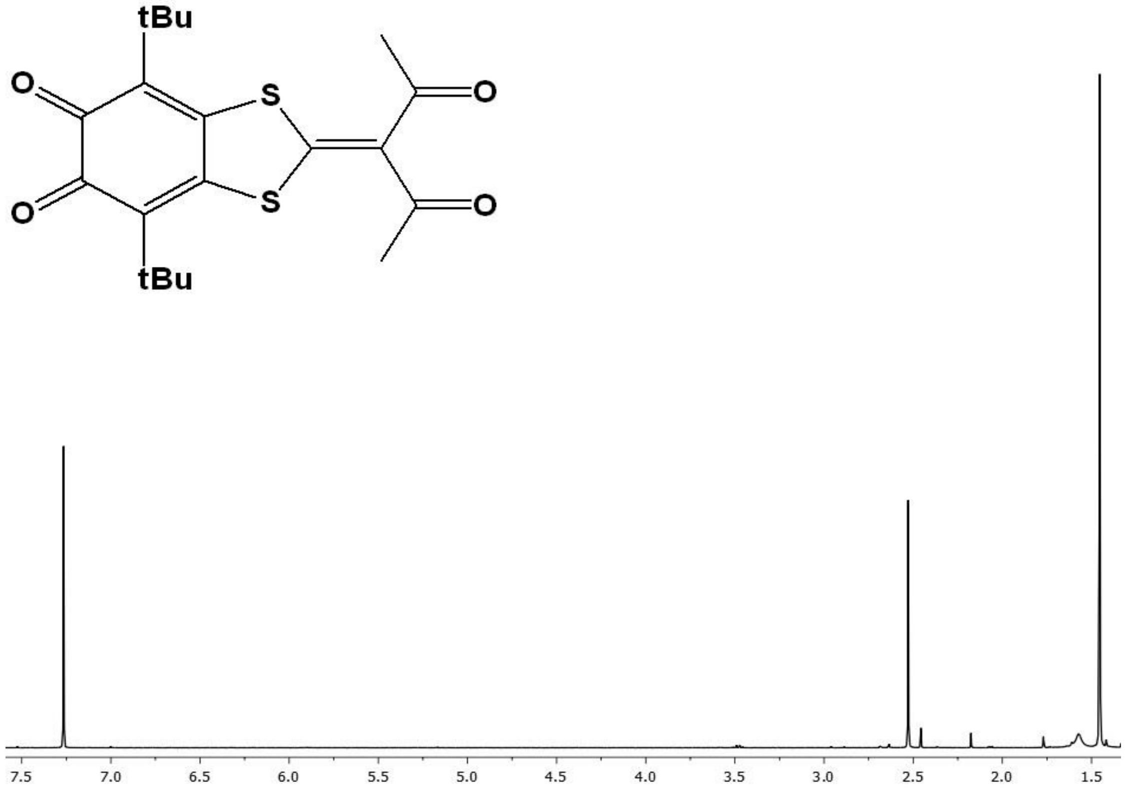

Figure S48. ^{1}H NMR spectrum of quinone **6d** in $CDCl_3$

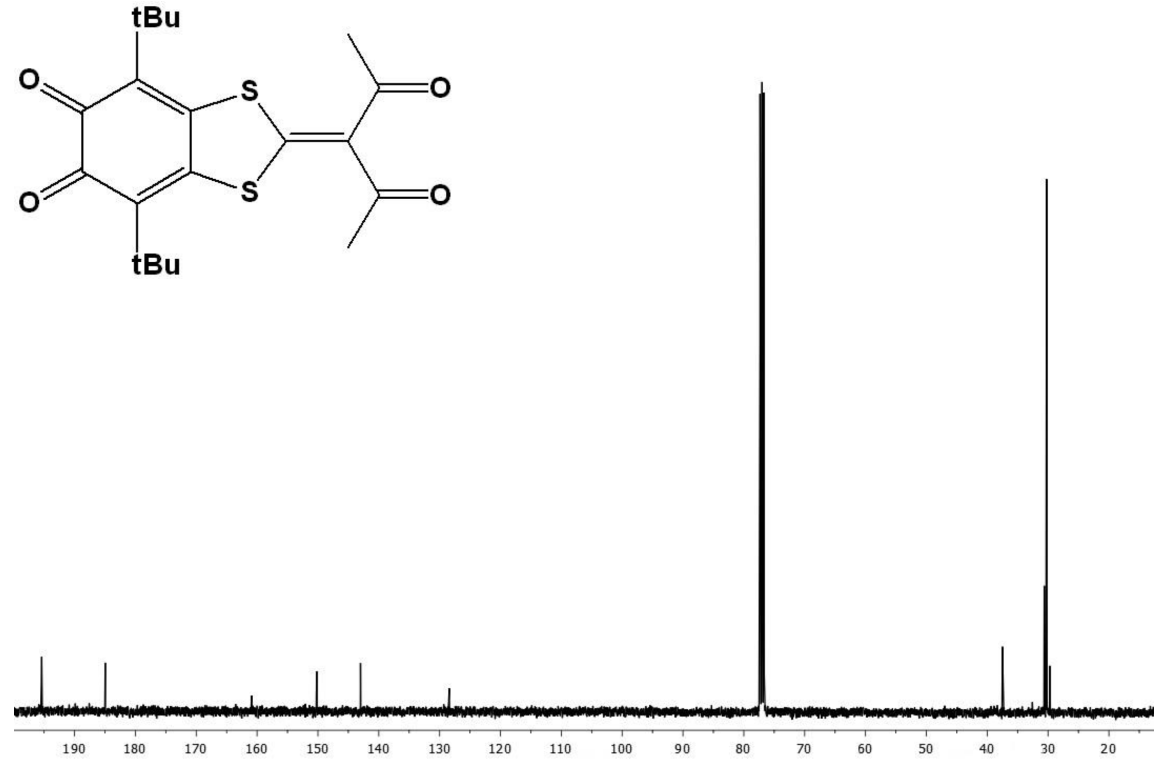

Figure S49. ^{13}C NMR spectrum of quinone **6d** in $CDCl_3$

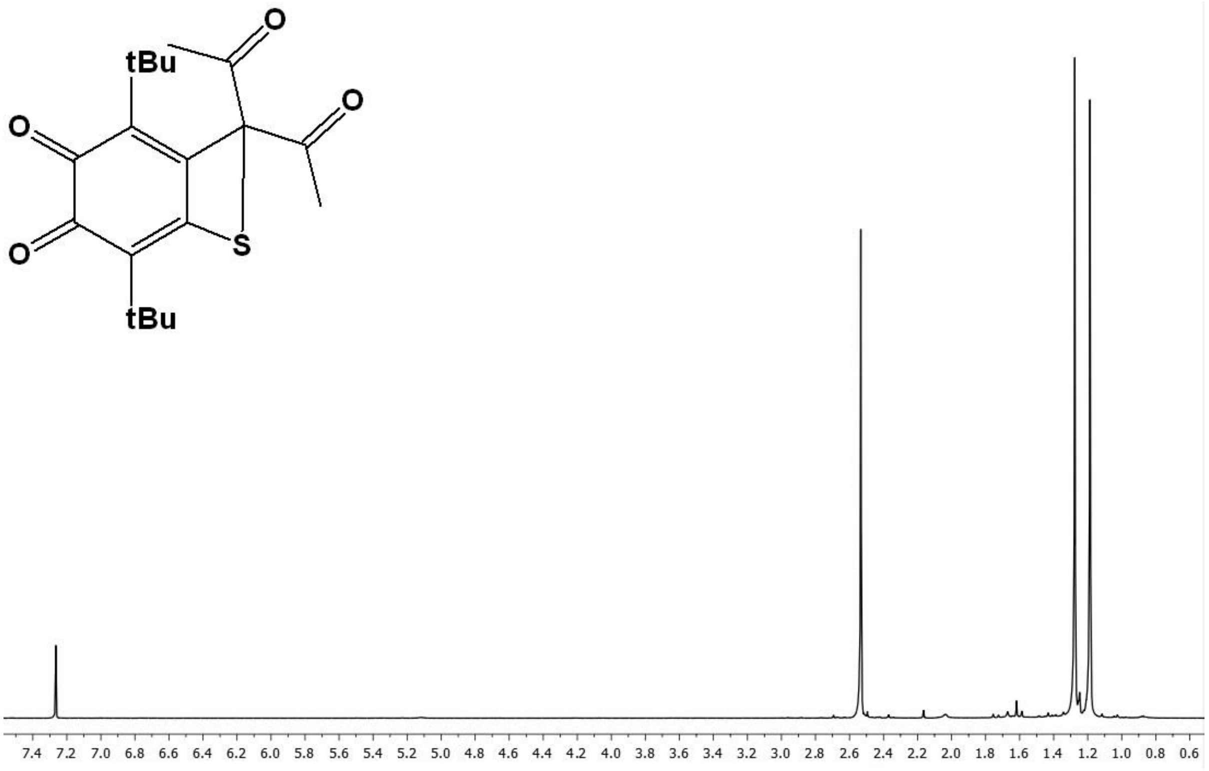

Figure S50. ^{1}H NMR spectrum of quinone **7** in $CDCl_3$

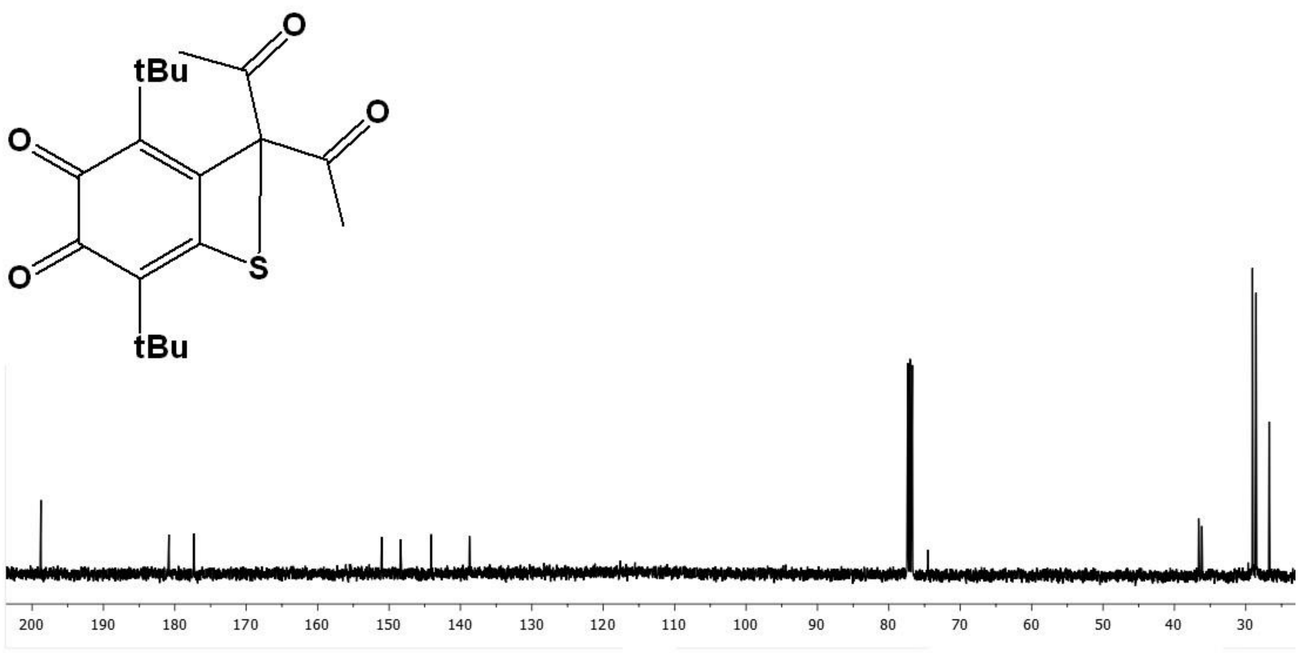

Figure S51. ^{13}C NMR spectrum of quinone **7** in $CDCl_3$

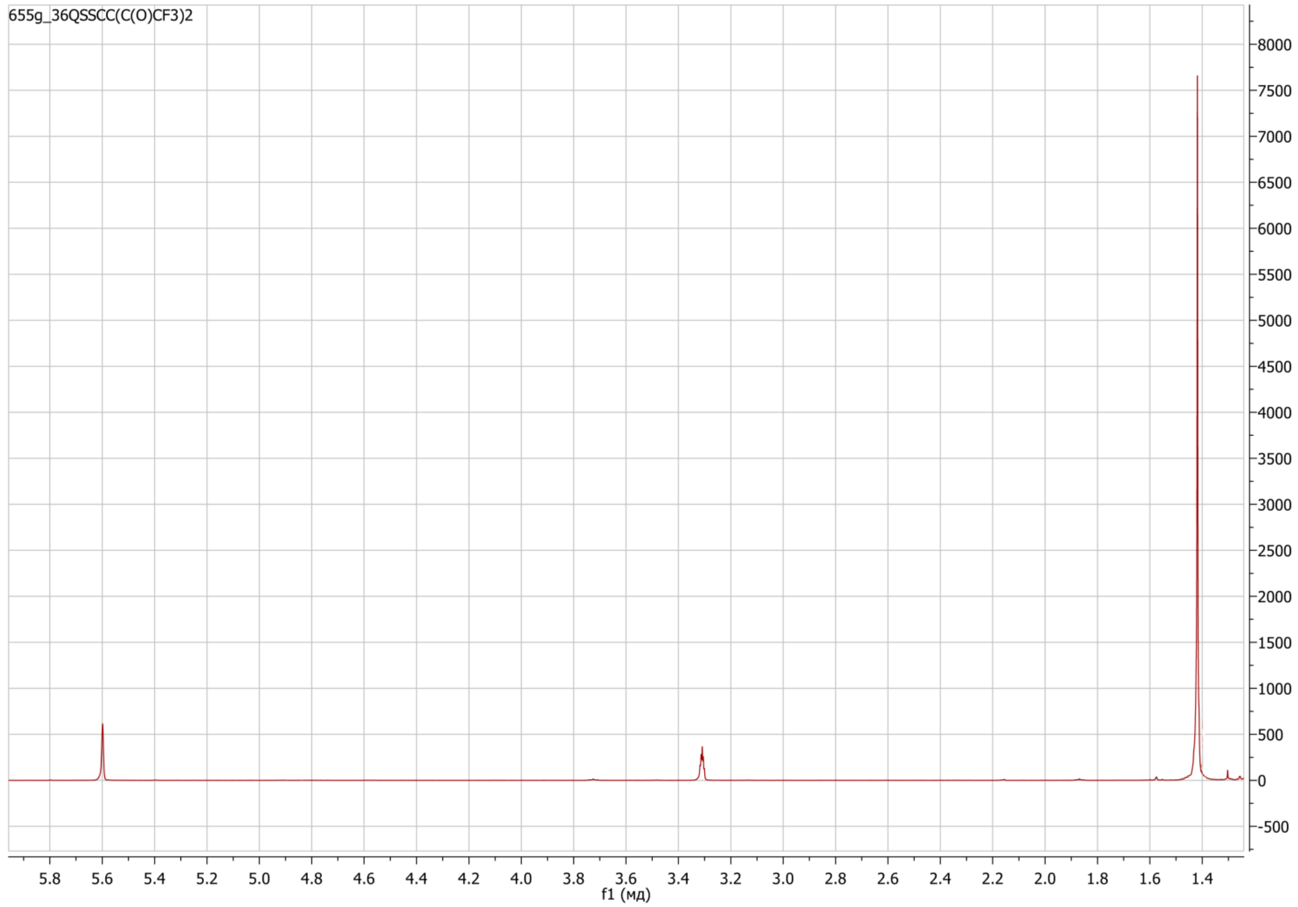

Figure S52. ^{1}H NMR spectrum of **8** in CD_3OD

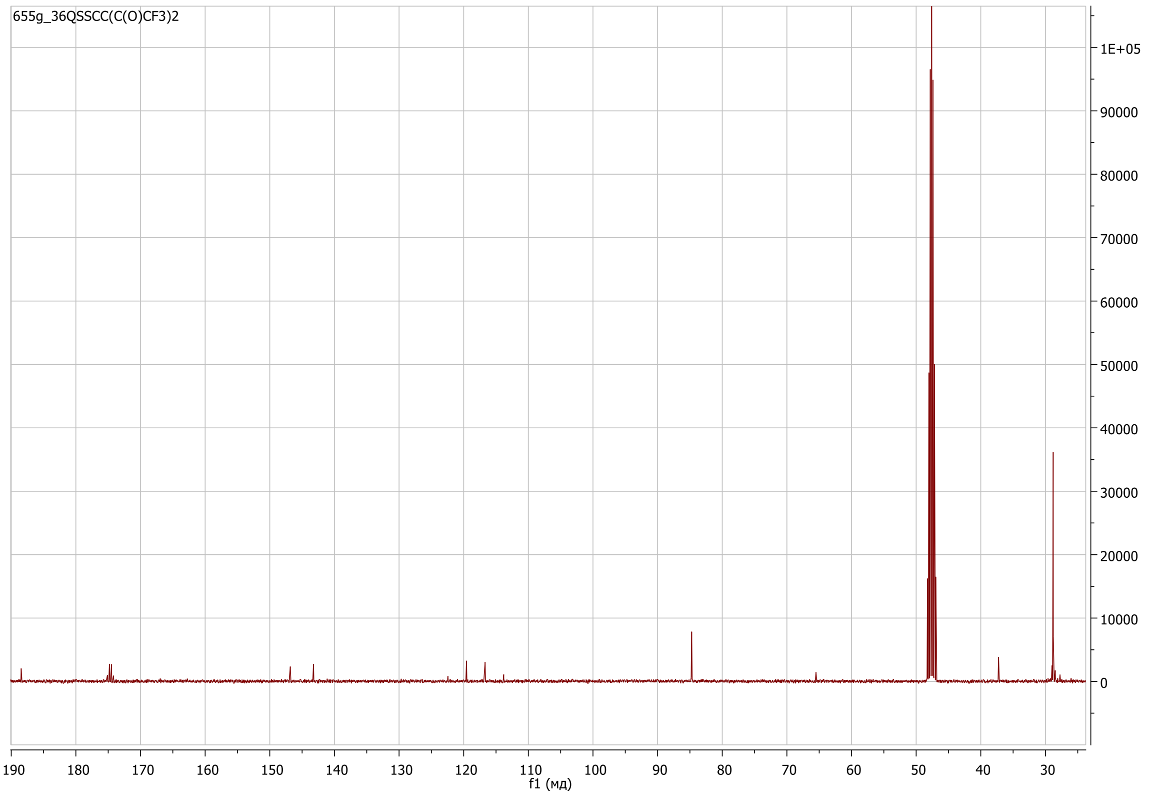

Figure S53. ^{13}C NMR spectrum of **8** in CD_3OD

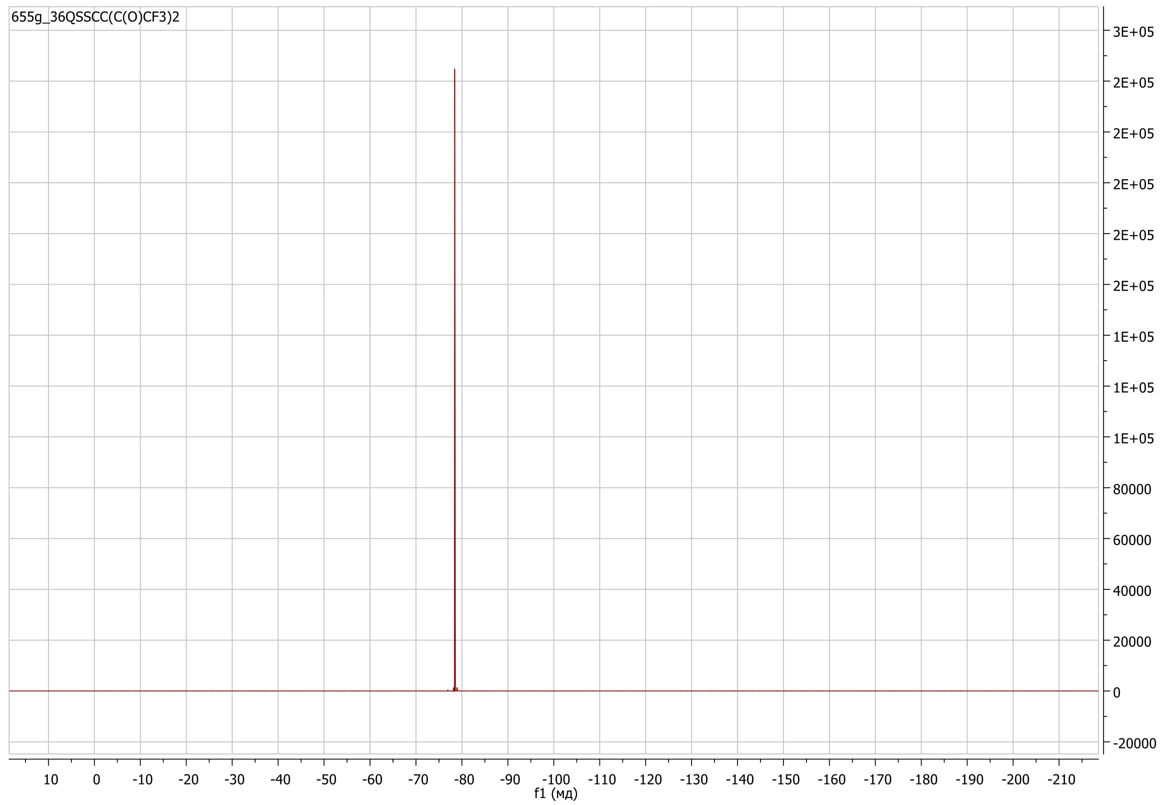

Figure S54. ^{19}F NMR spectrum of **8** in CD_3OD
